# Multi-objective optimization for 3D heterogeneous WSN deployment using an enhanced Genghis Khan shark algorithm

**DOI:** 10.1038/s41598-026-45399-z

**Published:** 2026-04-12

**Authors:** Essam H. Houssein, Ibrahim E. Ibrahim, Yaser M. Wazery, Marwa M. Emam

**Affiliations:** 1 Faculty of Computers and Information, Minia University, Minia, Egypt; 2 Minia National University, Minia, Egypt; 3 Faculty of Computers and Information, Luxor University, Luxor, Egypt

**Keywords:** Wireless sensor network, Wireless sensor network deployment, Connectivity, Multi-objective, Optimization, Energy science and technology, Engineering, Mathematics and computing

## Abstract

Heterogeneous three-dimensional (3D) wireless sensor network (WSN) deployment requires balancing sensing coverage, communication connectivity, and deployment cost under coupled *K*-coverage and *C*-connectivity constraints. This setting yields a constrained mixed discrete optimization landscape where many conventional multi-objective methods lose diversity or handle feasibility inconsistently. We formulate heterogeneous 3D WSN deployment as a constrained multi-objective problem and propose the Enhanced Multi-Objective Genghis Khan Shark Optimizer (EnMOGKSO). The core novelty is the integration of leader-pursuit dynamics with (i) dual archive-guided selection (elite and neighborhood memories), (ii) bounded external archive diversity control, and (iii) feasibility-first environmental selection for fragmented feasible regions. On the Congress on Evolutionary Computation (CEC) 2020 suite, EnMOGKSO obtains the best Friedman mean ranks in hypervolume (HV) (2.04) and inverted generational distance (IGD) (2.38), with statistically significant differences against most competitors ($p<0.05$, Wilcoxon/Friedman). In heterogeneous 3D WSN deployment, EnMOGKSO yields higher coverage/connectivity values (typically coverage means around 11–12 and connectivity around 7) than weaker baselines (often coverage 5–7 and connectivity 4–5), with higher but stable deployment cost. Overall, the results indicate a stronger convergence-diversity balance and more reliable feasibility-aware search under tight constraints, with practical applicability to 3D monitoring tasks such as industrial facilities, smart buildings, and environmental sensing.

## Introduction

Wireless sensor networks (WSNs) are increasingly deployed in volumetric and heterogeneous environments, including urban infrastructure, multi-story buildings, forest canopies, and industrial facilities. In such settings, planar (2D) assumptions are often insufficient for modeling sensing and communication. Deployment must therefore be optimized in three-dimensional (3D) space while accounting for heterogeneity in sensing range, communication radius, and deployment cost.

We consider the *heterogeneous 3D WSN deployment problem*, where sensor locations and sensor types are jointly selected to (i) maximize sensing coverage, (ii) maintain reliable multi-hop connectivity, and (iii) satisfy cost constraints. These objectives are inherently conflicting: improving coverage often requires additional nodes or larger sensing radii, both of which increase cost. Enforcing stronger connectivity may also induce spatial clustering, which can reduce coverage efficiency. In 3D settings, connectivity depends on volumetric inter-node distances, while heterogeneity introduces discrete type-selection decisions in addition to spatial placement. The resulting optimization problem is constrained, nonlinear, combinatorial, and NP-hard in practical formulations, making exact methods impractical at realistic scales.

Differences in sensing radius, communication behavior, and installation cost change both objective values and feasibility boundaries. Consequently, homogeneous approximations can yield overly optimistic deployment plans that violate practical connectivity or budget constraints once real sensor classes are used. This motivates explicit heterogeneous modeling in both the decision space and constraint set.

The deployment formulation combines mixed decision variables, non-convex objective interactions, and fragmented feasible regions under coupled *K*-coverage and *C*-connectivity constraints. Under these conditions, deterministic methods are difficult to scale, while Pareto metaheuristics provide a practical way to recover multiple high-quality trade-off deployments in a single optimization run.

Multi-objective evolutionary and swarm-based algorithms are natural candidates for this task because they approximate trade-off sets within a single run. However, under heterogeneous 3D deployment with mixed discrete–continuous variables and strict feasibility constraints, conventional Pareto-based methods (e.g., the Nondominated Sorting Genetic Algorithm II (NSGA-II), Multi-Objective Particle Swarm Optimization (MOPSO), and decomposition-based approaches) often exhibit premature convergence, archive crowding, and unstable feasibility behavior in sparse feasible regions. In addition, generic operators do not explicitly exploit deployment structure, where small spatial perturbations can trigger discontinuous changes in coverage or connectivity.

To address these limitations, we introduce the *Enhanced Multi-Objective Genghis Khan Shark Optimizer (EnMOGKSO)* for heterogeneous 3D WSN deployment. EnMOGKSO integrates leader-pursuit swarm dynamics with Pareto-archive guidance and constraint-domination selection to preserve diversity and improve feasibility-aware search in discontinuous deployment landscapes. Unlike generic multi-objective evolutionary algorithms (MOEAs), the framework explicitly couples mixed encoding (3D positions and sensor types) with archive-driven diversity control, enabling exploration of constrained trade-off regions while preserving structural feasibility.

The main contributions are as follows:

- We formulate heterogeneous 3D WSN deployment as a constrained multi-objective optimization problem that jointly models sensing coverage, communication connectivity, and deployment cost using unified notation.
- We develop EnMOGKSO as a multi-objective extension of GKSO, combining leader-pursuit dynamics, dual-archive management, and feasibility-first constraint domination to improve convergence stability and diversity preservation.
- We design a mixed encoding-decoding strategy for joint optimization of 3D placement and sensor-type selection, with explicit mapping between search operators and WSN objectives/constraints.
- We validate the method on CEC 2020 and heterogeneous 3D WSN scenarios using HV, IGD, and PSP with Wilcoxon/Friedman tests, with the base station fixed outside the deployment area.

The remainder of the paper is organized as follows. “Literature review” reviews related work and positions the proposed method. “Proposed EnMOGKSO and mathematical foundations” details EnMOGKSO and its mathematical foundations. “WSN system model and problem formulation” presents the WSN system model and formal problem formulation. “Experimental evaluation of EnMOGKSO” reports benchmark validation, and “Optimal WSN deployment results” reports heterogeneous WSN deployment results. “Conclusion and future work” concludes the paper.

## Literature review

This section reviews prior work on heterogeneous 3D WSN deployment in four categories: deployment geometry (2D/3D), objective modeling (energy/coverage/connectivity/cost), optimization strategies, and constraint handling. Across these categories, a recurring limitation is weak feasibility-aware diversity control when *K*-coverage and *C*-connectivity are enforced jointly.

### Review of WSN deployment strategies in 2D vs. 3D environments

WSN deployment research has progressed from simplified 2D assumptions to heterogeneous 3D settings that account for terrain and obstacle effects^1–3^. Recent studies increasingly combine domain-aware modeling with metaheuristic search to optimize coverage, connectivity, and cost simultaneously^4–6^.

Comparable trends are observed in underwater and irregular environments, where hybrid strategies can improve practical performance^7–14^. However, many methods still degrade when feasible regions are fragmented or objective conflicts are severe, especially in heterogeneous 3D deployment.

### Energy constraints

Energy optimization in WSNs has been studied through protocol design, routing, clustering, energy harvesting, and AI-assisted control^15–38^.

Despite this progress, energy behavior remains strongly coupled with deployment feasibility and multi-objective trade-offs, motivating methods that optimize coverage, connectivity, and cost jointly under constraints.

### Coverage models: k-coverage and its importance

Coverage models typically target robust *k*-coverage under energy and redundancy limits, often with hybrid metaheuristics^30,39–41^. Remaining challenges include scalability and stable solution quality under tight feasibility constraints.

### Connectivity models: C-connectivity and fault tolerance

Connectivity research spans clustering, relay placement, fault recovery, and learning-based adaptation^42–50^. These approaches improve reliability, but strict *C*-connectivity combined with simultaneous coverage/cost optimization remains challenging in heterogeneous 3D deployment.

### Cost model

Cost-aware deployment studies emphasize low-power hardware and platform efficiency to reduce installation and maintenance burden^51,52^. In practice, cost must be optimized jointly with coverage and connectivity constraints to avoid low-quality network designs.

### Existing optimization techniques

Deterministic optimization can provide exact solutions under restrictive assumptions, but it is generally impractical for large, nonlinear, and dynamic WSN deployment problems^53^. As a result, most current methods rely on metaheuristic and swarm-based approaches (e.g., GA, PSO, ACO, DE, WOA, GWO) for scalable search in constrained spaces^54,55^.

Multi-objective frameworks (e.g., NSGA-II, the Multi-Objective Evolutionary Algorithm based on Decomposition (MOEA/D), SPEA2, and hybrids) are now standard for balancing coverage, connectivity, energy, and cost^50,56^. However, diversity preservation and stable feasibility handling remain difficult in heterogeneous 3D settings. Table 1 summarizes representative methods and their limitations. Relative to this literature, EnMOGKSO is positioned as a feasibility-aware, dual-archive swarm framework for constrained heterogeneous 3D deployment.

**Table 1:** Review of literature: coverage, connectivity, and cost optimizations.

Ref	Objectives				2-D/3-D	Methodology/limitations
	Conn.	Cov.	Cost	Other		
^35^	$\checkmark$	$\checkmark$	$\checkmark$	Scalability	2-D	Heuristic Algorithm: Limited scalability in heterogeneous environments.
^16^	$\checkmark$	$\checkmark$	$\checkmark$	Sustainability	2-D	Heuristic Algorithm: Increased computational costs for large-scale deployments.
^17^	$\checkmark$	$\checkmark$	$\checkmark$	Energy Efficiency	2-D	Machine Learning: Relies on high-quality data for accurate predictions.
^18^	$\checkmark$	X	$\checkmark$	Energy Savings	3-D	Nano-Technology: High implementation costs due to advanced hardware.
^19^	X	$\checkmark$	$\checkmark$	Reliability	2-D	Heuristic Algorithm: Limited energy optimization for large-scale logistics networks.
^20^	X	$\checkmark$	$\checkmark$	Decentralized Energy	2-D	Blockchain: High overhead due to blockchain transaction costs.
^21^	X	$\checkmark$	$\checkmark$	Sustainability	3-D	RF Energy Harvesting: Requires reliable energy sources for consistent performance.
^22^	$\checkmark$	$\checkmark$	X	Scalability	3-D	Bayesian Optimization: Computational complexity in dynamic aerial environments.
^23^	$\checkmark$	$\checkmark$	$\checkmark$	Cost Efficiency	2-D	Heuristic Algorithm: Limited range and data rate for large-scale monitoring.
^24^	$\checkmark$	X	$\checkmark$	Adaptability	3-D	Evolutionary Game Theory: High energy consumption for UAVs in long-duration tasks.
^25^	$\checkmark$	X	X	Latency Reduction	2-D	Adaptive MAC Protocol: Inefficient for sparse deployments with low traffic.
^26^	$\checkmark$	X	X	Reliability	2-D	Adaptive Backoff Algorithm: Performance decreases with increasing network density.
^27^	$\checkmark$	X	X	Collision Resolution	3-D	Game Theory-Based Protocol: Not suitable for highly dynamic underwater environments.
^28^	$\checkmark$	X	X	Load Balancing	2-D	Swarm Intelligence: High computational cost for large-scale networks.
^29^	$\checkmark$	X	X	Scalability	2-D	Hybrid Swarm Optimization: Limited adaptability in real-time topology changes.
^30^	$\checkmark$	$\checkmark$	X	Network Awareness	2-D	Deep Reinforcement Learning: Training requires significant computational resources.
^31^	$\checkmark$	X	X	Reliability	2-D	Joint Routing and Storage: Limited performance in highly dynamic environments.
^32^	$\checkmark$	$\checkmark$	$\checkmark$	Energy Efficiency	2-D	Stochastic PSO: Convergence speed is slow for complex networks.
^39^	$\checkmark$	$\checkmark$	X	Adaptability	2-D	Ant Colony Optimization: High convergence time in large-scale scenarios.
^41^	$\checkmark$	$\checkmark$	X	Efficiency	2-D	Simulated Annealing: Computationally expensive for high-density deployments.
^42^	$\checkmark$	X	X	Robustness	2-D	Clustering Algorithm (LEACH): Frequent cluster reformation increases energy costs.
^57^	$\checkmark$	X	$\checkmark$	Scalability	2-D	Pareto-Based Optimization: High complexity in balancing multiple objectives.
^46^	$\checkmark$	$\checkmark$	X	Scalability	3-D	Heterogeneous Resource Allocation: Increased computational overhead in heterogeneous deployments.
^49^	$\checkmark$	X	X	Connectivity Restoration	3-D	AI Restoration Techniques: Requires pre-trained models for effective restoration.

## Proposed EnMOGKSO and mathematical foundations

### Genghis Khan shark optimizer (GKSO)

GKSO is a swarm metaheuristic inspired by hunting and survival behaviors. Its search process contains four components: exploration, exploitation, foraging transition, and defense^58^.

#### Wandering hunting stage (exploration)

This stage samples broad candidate positions within variable bounds to emphasize global exploration.

$$\begin{aligned} \begin{aligned} X_i^j(t+1)&= X_i^j(t) + \frac{lb_j + r_1 \cdot (ub_j - lb_j)}{i \cdot t}, \\&\quad i = 1,2,\ldots ,N;\quad j = 1,2,\ldots ,D \end{aligned} \end{aligned}$$

where $r_1\in [0,1]$, *N* is population size, *D* is dimensionality, and *t* is the current iteration.

#### Moving towards the best hunting position (exploitation)

This stage moves each agent toward the best-known position, with attraction strength proportional to detected signal intensity:

1 $$\begin{aligned} \hat{X}_i^j(t+1)=\textrm{s}^*\left( X_{best }^j(t)-X_i^j(t)\right) \end{aligned}$$

with

2 $$\begin{aligned} \textrm{s} = m I^r \end{aligned}$$

where $r\in [0,1]$ is random, *I* is fitness-related intensity, and *m* is a control constant. The final update averages attraction and historical motion:

3 $$\begin{aligned} X_i^j(t+1)=\frac{\hat{X}_i^j(t+1)+X_{i-1}^j(t)}{2} \end{aligned}$$

#### Parabolic foraging

This phase models cooperative exploitation around the current best position:

4 $$\begin{aligned} \begin{aligned} X_i^j(t+1)&= X_{\text {best}}^j(t) + r_2 \cdot \left( X_{\text {best}}^j(t) - X_i^j(t)\right) \\&\quad + \lambda \cdot p^2 \cdot \left( X_{\text {best}}^j(t) - X_i^j(t)\right) \end{aligned} \end{aligned}$$

where *p* controls exploration-exploitation step size over iterations:

5 $$\begin{aligned} p=2 *\left\{ 1-\left( \frac{t}{T}\right) ^{\frac{1}{4}}+|\omega (t+1)| *\left[ \left( \frac{t}{T}\right) ^{\frac{1}{4}}-\left( \frac{t}{T}\right) ^3\right] \right\} . \end{aligned}$$

where $|\omega (\textrm{t}+1)|$ denotes the weight coefficient at time $t+1$ and is computed as follows:

6 $$\begin{aligned} |\omega (t+1)|=1-2 \omega ^4(t) \end{aligned}$$

#### Self protection mechanism

This stage introduces defensive perturbations around elite solutions to diversify local search and avoid stagnation.

7 $$\begin{aligned} X_i^j(t+1) = \left\{ \begin{aligned}&X_i^j(t) + k_1\left( a_1 X_{\text {best}}^j(t) - a_2 X_k^j(t)\right) + k_2 \rho \left( a_3\left( X_{2_i}^j(t) - X_{1_i}^j(t)\right) \right) \\&\quad + \dfrac{a_2}{2} \left( X_{u1}(t) - X_{u2}(t)\right) , & \text {if } a_1 < 0.5 \\&X_{\text {best}}^j(t) + k_1\left( a_1 X_{\text {best}}^j(t) - a_2 X_k^j(t)\right) + k_2 \rho \left( a_3\left( X_{2_i}^j(t) - X_{1_i}^j(t)\right) \right) \\&\quad + \dfrac{a_2}{2} \left( X_{u1}(t) - X_{u2}(t)\right) , & \text {otherwise} \end{aligned} \right. \end{aligned}$$

where $k_1\in [-1,1]$, $k_2\sim \mathcal {N}(0,1)$, and $a_1,a_2,a_3$ are random coefficients.

8 $$\begin{aligned} X_k^j(t)=l_2^*\left( X_p^j(t)-X_r^j(t)\right) +X_r^j(t) \end{aligned}$$

Here, $X_r^j(t)$ is a random solution, $X_p^j(t)$ is a sampled population member, and $\rho$ is an adaptive coefficient:

9 $$\begin{aligned} \rho =\alpha ^*\left( 2^* \text{ rand } -1\right) \end{aligned}$$

10 $$\begin{aligned} \alpha =\, \beta ^* \sin \left( \frac{3 \pi }{2}+\sin \left( \frac{3 \pi }{2} \beta \right) \right) \end{aligned}$$

11 $$\begin{aligned} \beta =\beta _{\min }+\left( \beta _{\max }-\beta _{\min }\right) *\left( 1-\left( \frac{i t}{T}\right) ^3\right) ^2 \end{aligned}$$

This mechanism perturbs candidates around the current best and keeps the strongest trial, improving local escape and refinement.

### Basic concepts of multi-objective optimization problems (MOPs)

Multi-objective optimization addresses problems with conflicting objectives and seeks a set of non-dominated trade-off solutions rather than a single optimum.

#### Mathematical formulation

A general MOP is defined by an objective vector and a feasible set. For consistency, we use the standard minimization form.

12 $$\begin{aligned} \begin{aligned}&\min \ {\bf f}({\bf x}) = \left( f_1({\bf x}), f_2({\bf x}), \ldots , f_m({\bf x})\right) \\&\text {s.t.}\ {\bf x}\in \Omega . \end{aligned} \end{aligned}$$

#### Pareto optimality

Pareto dominance is used to compare conflicting objective vectors and define non-dominated trade-off solutions.

A solution ${\bf x}_1 \in \Omega$ is said to *dominate* another solution ${\bf x}_2 \in \Omega$, denoted ${\bf x}_1 \prec {\bf x}_2$, if and only if the following two conditions are satisfied:

13 $$\begin{aligned} \begin{aligned}&\forall i \in \{1, ..., m\},\; f_i({\bf x}_1) \le f_i({\bf x}_2) \\&\quad \text {and} \quad \exists j \in \{1, ..., m\} \text { such that } f_j({\bf x}_1) < f_j({\bf x}_2) \end{aligned} \end{aligned}$$

A solution is Pareto optimal if no other feasible solution dominates it. The corresponding non-dominated set in decision space is the Pareto set (PS), and its image in objective space is the Pareto front (PF).

## WSN system model and problem formulation

The deployment problem is defined over a finite set of 3D candidate locations and heterogeneous sensor types, with three coupled criteria: sensing coverage, network connectivity, and deployment cost. We first define decision variables, then present compact sensing and communication models, and finally state the constrained multi-objective formulation.

### Deployment domain, targets, and sensor types

Let $\mathcal {P}=\{{\bf p}_1,\ldots ,{\bf p}_{N_{\textrm{loc}}}\}$ denote the set of candidate deployment locations, where ${\bf p}_i=(x_i,y_i,z_i)\in \mathbb {R}^3$. Let $\mathcal {T}=\{\tau _1,\ldots ,\tau _{N_{\textrm{tgt}}}\}$ denote the set of target points, $\tau _j\in \mathbb {R}^3$. Sensors belong to $N_{\textrm{cls}}$ classes indexed by $\kappa \in \{1,\ldots ,N_{\textrm{cls}}\}$; class $\kappa$ has sensing radius $r(\kappa )$ and unit cost $\xi (\kappa )$. Each candidate site *i* has a location-dependent deployment factor $\zeta (i)>0$.

*Decision variables.* Let $\chi _i^{\kappa }\in \{0,1\}$ indicate whether a sensor of class $\kappa$ is deployed at site ${\bf p}_i$. At most one sensor can be deployed at each site:

14 $$\begin{aligned} \sum _{\kappa =1}^{N_{\textrm{cls}}}\chi _i^{\kappa }\le 1,\quad \forall i\in \{1,\ldots ,N_{\textrm{loc}}\}. \end{aligned}$$

### Notation summary

Table 2 lists the main symbols used in the WSN model and deployment formulation.

**Table 2:** Notation used in the WSN system model and deployment formulation.

Symbol	Description
$\mathcal {P}$, $N_{\textrm{loc}}$	Candidate deployment sites and their count
$\mathcal {T}$, $N_{\textrm{tgt}}$	Target points and their count
$N_{\textrm{cls}}$	Number of sensor classes
$\chi _i^{\kappa }$	Binary decision variable (place class $\kappa$ at site *i*)
$r(\kappa )$, $\xi (\kappa )$	Sensing radius and unit cost of class $\kappa$
$\zeta (i)$	Location-dependent deployment cost factor at site *i*
*K*, *C*	Minimum coverage multiplicity and nodal connectivity thresholds
$f_{\textrm{cost}}, f_{\textrm{cov}}, f_{\textrm{conn}}$	Cost, coverage, and connectivity objectives
$v(\chi )$	Total constraint violation
$\tau$, $a_i$	Decoder threshold and site activation indicator
$r_c,r_e,\lambda _1,\lambda _2$, $\theta$	Parameters of the probabilistic link model

### Coverage model and K-coverage constraint

For a target $\tau _j$ and site ${\bf p}_i$, define the sensing indicator

15 $$\begin{aligned} \gamma ({\bf p}_i,\tau _j,\kappa )= {\left\{ \begin{array}{ll} 1, & \Vert {\bf p}_i-\tau _j\Vert _2\le r(\kappa ),\\ 0, & \text {otherwise.} \end{array}\right. } \end{aligned}$$

The coverage multiplicity of target $\tau _j$ is

16 $$\begin{aligned} m_{\tau _j}=\sum _{i=1}^{N_{\textrm{loc}}}\sum _{\kappa =1}^{N_{\textrm{cls}}}\gamma ({\bf p}_i,\tau _j,\kappa )\chi _i^{\kappa }. \end{aligned}$$

We enforce *K*-coverage for every target:

17 $$\begin{aligned} m_{\tau _j}\ge K,\quad \forall j\in \{1,\ldots ,N_{\textrm{tgt}}\}. \end{aligned}$$

As a scalar coverage objective, we use the average coverage multiplicity:

18 $$\begin{aligned} f_{\textrm{cov}}(\chi )=\mu _{\textrm{cov}}=\frac{1}{N_{\textrm{tgt}}}\sum _{j=1}^{N_{\textrm{tgt}}}m_{\tau _j}. \end{aligned}$$

### Communication/connectivity model and C-connectivity constraint

We adopt a probabilistic communication model between two (candidate) locations ${\bf p}_i$ and ${\bf p}_j$:

19 $$\begin{aligned} \psi ({\bf p}_i,{\bf p}_j)= {\left\{ \begin{array}{ll} 1, & \Vert {\bf p}_i-{\bf p}_j\Vert _2\le r_c-r_e,\\ \exp \!\left( -\dfrac{\lambda _1}{b^{\lambda _2}}\right) , & \left| \Vert {\bf p}_i-{\bf p}_j\Vert _2-r_c\right| < r_e,\\ 0, & \text {otherwise,} \end{array}\right. } \end{aligned}$$

where $b=\Vert {\bf p}_i-{\bf p}_j\Vert _2-(r_c-r_e)$ and $(r_c,r_e,\lambda _1,\lambda _2)$ are communication-model parameters. To obtain a deterministic adjacency relation, we define the binary link indicator

20 $$\begin{aligned} \eta ({\bf p}_i,{\bf p}_j)= {\left\{ \begin{array}{ll} 1, & \psi ({\bf p}_i,{\bf p}_j)\ge \theta ,\\ 0, & \text {otherwise,} \end{array}\right. } \end{aligned}$$

where $\theta \in [0,1]$ is a connectivity threshold.

The nodal connection degree (number of neighbors) of site *i* induced by a deployment $\chi$ is defined as

21 $$\begin{aligned} c_{\textrm{conn},i}=\sum _{\begin{array}{c} j=1\\ j\ne i \end{array}}^{N_{\textrm{loc}}}\eta ({\bf p}_i,{\bf p}_j)\sum _{\kappa =1}^{N_{\textrm{cls}}}\chi _j^{\kappa }. \end{aligned}$$

We impose a minimum *C*-connectivity requirement for each deployed node using a standard activation trick:

22 $$\begin{aligned} c_{\textrm{conn},i}\ge C\cdot \sum _{\kappa =1}^{N_{\textrm{cls}}}\chi _i^{\kappa },\quad \forall i\in \{1,\ldots ,N_{\textrm{loc}}\}. \end{aligned}$$

As a scalar connectivity objective, we report the average nodal degree over deployed sensors:

23 $$\begin{aligned} f_{\textrm{conn}}(\chi )=\mu _{\textrm{conn}}=\frac{\sum _{i=1}^{N_{\textrm{loc}}}c_{\textrm{conn},i}}{\sum _{i=1}^{N_{\textrm{loc}}}\sum _{\kappa =1}^{N_{\textrm{cls}}}\chi _i^{\kappa }}. \end{aligned}$$

### Cost model

The total deployment cost combines the sensor hardware cost and the location-dependent deployment factor:

24 $$\begin{aligned} f_{\textrm{cost}}(\chi )=C_{\textrm{total}}=\sum _{i=1}^{N_{\textrm{loc}}}\sum _{\kappa =1}^{N_{\textrm{cls}}}\xi (\kappa )\zeta (i)\chi _i^{\kappa }. \end{aligned}$$

Unlike uniform-cost assumptions, $\zeta (i)$ captures spatial differences in accessibility, installation effort, and site preparation burden. This improves deployment realism in irregular terrains and built environments where physically difficult locations induce higher practical cost.

### Multi-objective deployment optimization problem

The deployment of heterogeneous sensors is formulated as a multi-objective optimization problem that jointly considers several conflicting design objectives. In practical wireless sensor network (WSN) deployments, improving sensing performance or network connectivity typically requires additional sensors or higher-capability devices, which increases deployment cost. Therefore, an appropriate deployment strategy must carefully balance these competing requirements.

We simultaneously (i) minimize deployment cost, (ii) maximize coverage multiplicity, and (iii) maximize connectivity degree, which together represent the key performance indicators of a reliable WSN deployment, subject to explicit *K*-coverage and *C*-connectivity constraints. To maintain consistency with Pareto-based multi-objective optimization methods, the problem is expressed in a minimization form. Consequently, the maximization objectives related to coverage and connectivity are transformed into minimization terms by taking their negative values. Using a minimization form consistent with Pareto dominance, the deployment problem is:

25 $$\begin{aligned} \begin{aligned}&\min _{\chi }\ \big (f_{\textrm{cost}}(\chi ),\ -f_{\textrm{cov}}(\chi ),\ -f_{\textrm{conn}}(\chi )\big )\\&\text {s.t.}\quad (14),\ (17),\ (22),\\&\qquad \ \chi _i^{\kappa }\in \{0,1\},\quad \forall i,\kappa . \end{aligned} \end{aligned}$$

In this formulation, the binary decision variable $\chi _i^{\kappa }$ indicates whether a sensor of class $\kappa$ is deployed at candidate location *i*.

The proposed formulation explicitly models sensor heterogeneity through the class-dependent deployment variable $\chi _i^{\kappa }$. This allows different sensor types to contribute differently to coverage performance, communication connectivity, and deployment cost. Such modeling reflects realistic deployment conditions where sensors may differ in sensing range, communication capability, and hardware cost.

Feasibility of candidate deployment solutions is guaranteed by enforcing the *K*-coverage and *C*-connectivity constraints, ensuring that every monitored location is covered by at least *K* sensors while maintaining sufficient network connectivity for reliable data transmission.

Accurate modeling of sensor heterogeneity is particularly important because homogeneous assumptions may significantly overestimate network feasibility. In practice, ignoring hardware diversity can result in deployment strategies that appear optimal in simulation but become difficult to implement due to physical device limitations or budget constraints.

## Experimental framework and statistical interpretation

This section presents the evaluation protocol for two tracks: CEC 2020 benchmarks and constrained heterogeneous 3D WSN deployment (“WSN system model and problem formulation”), with emphasis on reproducibility and statistical validity.

### Algorithms compared and common experimental settings

EnMOGKSO is compared with representative Pareto-based baselines (e.g., NSGA-II, MOPSO, MOGWO, MOEA/D) under a common budget (identical maximum function evaluations, run count, and stopping rule).

#### Randomization and reproducibility

Each algorithm is executed for *R* independent runs with different random seeds. Metrics are computed from the final non-dominated set of each run and summarized by mean/standard deviation (SD), best, and worst values.

### Parameter configuration

Table 3 reports the parameter settings for EnMOGKSO and the compared algorithms. To ensure fairness, values were adopted from source papers when available; otherwise, they were tuned on a small validation subset and then fixed for all tests.

**Table 3:** Parameter settings used in the experiments (all algorithms share the same population size and evaluation budget unless stated otherwise).

Component	Symbol / name	Setting / description
Common	Population size	$N=100$
Common	Iteration budget	$MaxIt=500$
Common	Independent runs	$R=20$
EnMOGKSO	External archive size	$A=100$ (maximum number of non-dominated solutions retained)
EnMOGKSO	Dual-archive design	PBA (elite) + NBA (diversity/novelty) with pruning by crowding/density
EnMOGKSO	Leader selection	Diversity-aware leader sampling from PBA/NBA (probability biased to sparse regions)
EnMOGKSO	Constraint handling	Feasibility-first dominance + penalty/repair for constraint violations
WSN model	Coverage requirement	*K*-coverage constraint in (17)
WSN model	Connectivity requirement	*C*-connectivity constraint in (22)
WSN model	Link threshold	$\theta$ in (20)
WSN model	Comm. parameters	$(r_c,r_e,\lambda _1,\lambda _2)$ in (19)

All algorithms are run under the same common budget for fair comparison.

### Scenario descriptions

#### CEC 2020 multi-objective benchmark scenarios

The CEC 2020 suite contains 24 multi-objective test functions with varying Pareto-front shapes, separability, modality, and variable interactions. These properties diagnose optimizer behavior: multimodality stresses global exploration, nonseparability stresses coordinated high-dimensional search, and irregular fronts test archive-level diversity maintenance. For each function, we follow the standard suite settings (dimension and objective count) and evaluate all algorithms under the same evaluation budget.

#### 3D heterogeneous WSN deployment scenarios

We consider a 3D monitoring volume with candidate deployment sites $\mathcal {P}$ and target points $\mathcal {T}$. Each site can host at most one sensor type (constraint (14)). The objectives are cost minimization (24) and maximization of average coverage multiplicity (18) and average nodal degree (23), subject to *K*-coverage (17) and *C*-connectivity (22).

#### Scenario factors

To analyze scalability and constraint difficulty, scenarios systematically vary: (i) the number of targets $N_{\textrm{tgt}}$ (coverage density), (ii) the number of candidate sites $N_{\textrm{loc}}$ (search space size), (iii) heterogeneity level $N_{\textrm{cls}}$ (type-choice combinatorics), and (iv) constraints (*K*, *C*) (feasibility tightness). In all WSN scenarios, the base station (BS) is placed outside the deployment area. Under tight constraints (large *K* and/or *C*), feasible regions become fragmented; effective optimizers must preserve feasible candidates while exploring trade-offs.

### Performance indicators and metric definitions

We use three standard multi-objective quality indicators to quantify convergence and diversity of the approximation set *P* relative to a reference Pareto set $P^\star$.

#### Hypervolume (HV)

HV measures the dominated volume between the approximation set *P* and a user-defined reference point ${\bf r}$ in objective space. For minimization objectives, a larger HV indicates a better combination of proximity to the Pareto front and coverage/spread along the front:

26 $$\begin{aligned} \textrm{HV}(P)=\Lambda \left( \bigcup _{{\bf f}\in P} [f_1, r_1]\times \cdots \times [f_M, r_M] \right) , \end{aligned}$$

where $\Lambda (\cdot )$ is the Lebesgue measure and *M* is the number of objectives.

#### Inverted generational distance (IGD)

IGD measures the average distance from each point on the reference front to the approximation set:

27 $$\begin{aligned} \textrm{IGD}(P,P^\star )=\frac{1}{|P^\star |}\sum _{{\bf z}\in P^\star }\min _{{\bf f}\in P}\Vert {\bf z}-{\bf f}\Vert _2. \end{aligned}$$

Smaller IGD indicates that the approximation set is both close to and well distributed along the reference front.

#### Pareto set proximity (PSP)

PSP measures how close the obtained solutions are to the reference set. In this study, PSP is computed as the average distance from solutions in *P* to their nearest counterparts in $P^\star$:

28 $$\begin{aligned} \textrm{PSP}(P,P^\star )=\frac{1}{|P|}\sum _{{\bf f}\in P}\min _{{\bf z}\in P^\star }\Vert {\bf f}-{\bf z}\Vert _2. \end{aligned}$$

Smaller PSP suggests stronger convergence toward the true Pareto-optimal set.

The HV indicator quantifies both the dominance quality and the spread of the obtained Pareto solutions. In contrast, The IGD measures the average distance from the reference Pareto front to the set of solutions produced by the algorithm, thereby reflecting convergence and coverage of the true front. Meanwhile, the PSP metric evaluates the distance from the obtained solution set to the reference front, providing complementary information regarding the proximity of the generated solutions to the optimal Pareto boundary. Reporting these three indicators jointly mitigates the bias associated with relying on a single performance metric and enables a more balanced evaluation of optimization performance in terms of convergence, diversity, and approximation accuracy of the Pareto front.

### Statistical validation

Because indicator distributions are often non-normal and heteroscedastic, we use nonparametric tests.

#### Pairwise Wilcoxon signed-rank test

We compare EnMOGKSO with each competitor using the Wilcoxon signed-rank test ($\alpha =0.05$) on paired runs. One-sided alternatives are used: HV (higher is better) and IGD/PSP (lower is better).

#### Friedman test and ranking

Across all problems, the Friedman test ranks algorithms (rank 1 is best; HV descending, IGD/PSP ascending) and tests overall differences. When significant, mean ranks summarize global performance.

### Quantitative comparison protocol

For each CEC function and WSN scenario, we report mean, standard deviation, best, and worst values of HV/IGD/PSP over *R* runs. Best row values are highlighted, and statistical significance is indicated by Wilcoxon results.

### Scientific interpretation: why EnMOGKSO performs better

Relative to classical Pareto swarms, the observed gains are explained by four design choices that jointly improve diversity and feasibility in constrained mixed-variable search.

#### Mechanism 1: dual-archive selection reduces dominance pressure and prevents diversity collapse

EnMOGKSO uses an elite archive (PBA) and a novelty archive (NBA). PBA preserves high-quality solutions, while NBA preserves sparse or bridging solutions, improving front coverage (HV) and reducing missed regions (IGD).

#### Mechanism 2: feasibility-aware selection stabilizes search under tight K/C constraints

Feasibility-first dominance prioritizes feasible solutions and then lower violation among infeasible ones. This guides search toward feasible regions without collapsing diversity and improves robustness.

#### Mechanism 3: mixed representation benefits from continuous search plus deterministic decoding

A continuous score matrix is decoded into a discrete placement with “at most one sensor per site.” This keeps search smooth while enforcing combinatorial validity, reducing wasted evaluations and accelerating convergence.

#### Mechanism 4: leader sampling biased to sparse regions improves spread without sacrificing convergence

Density-aware leader sampling emphasizes under-represented regions, improving spread and hypervolume while keeping convergence competitive.

### Experimental summary

Results across CEC 2020 and heterogeneous 3D WSN scenarios indicate a better convergence-diversity balance and stronger feasibility handling, with nonparametric support from Wilcoxon and Friedman tests.

### Problem-specific encoding, decoding, and constraint handling

#### (A) Solution encoding

To enable the application of the continuous EnMOGKSO search operators to the discrete WSN deployment problem, each candidate solution (agent) is represented as a continuous vector that is later decoded into a feasible deployment configuration.

Each EnMOGKSO agent is a continuous vector $y\in \mathbb {R}^{D}$ with

29 $$\begin{aligned} D = |\mathcal {P}|\,|\mathcal {K}| = N_{\textrm{loc}}\,N_{\textrm{cls}}, \end{aligned}$$

where $\mathcal {K}=\{1,\ldots ,N_{\textrm{cls}}\}$ indexes heterogeneous sensor classes.

This representation allows the optimization algorithm to explore a continuous search space while still producing discrete deployment decisions after decoding.

We reshape *y* into a score matrix $Y\in \mathbb {R}^{N_{\textrm{loc}}\times N_{\textrm{cls}}}$, where $Y_{i\kappa }$ denotes the selection score of placing class $\kappa$ at site *i*.

Higher scores indicate stronger preference for selecting a particular sensor class at the corresponding deployment location.

The corresponding discrete deployment is represented by the binary placement variables $\chi _i^{\kappa }$ defined in “WSN system model and problem formulation”.

#### Continuous-to-binary transformation and “at most one sensor per site”

To transform the continuous score representation into a valid deployment configuration, a site-wise decoding rule is applied. This rule ensures that each candidate location hosts at most one sensor, while still allowing heterogeneous sensor types to be selected.

For each site *i*, we select a single sensor class (or none) by

30 $$\begin{aligned} \kappa _i^\star = \arg \max _{\kappa \in \mathcal {K}} Y_{i\kappa }, \qquad a_i = \mathbb {I}\big ( Y_{i\kappa _i^\star } \ge \tau \big ), \end{aligned}$$

and then set

31 $$\begin{aligned} \chi _i^{\kappa } = a_i\,\mathbb {I}(\kappa =\kappa _i^\star ),\quad \forall i,\kappa , \end{aligned}$$

where $\tau \in \mathbb {R}$ is an activation threshold and $\mathbb {I}(\cdot )$ is the indicator function.

The activation threshold $\tau$ controls whether a sensor is deployed at site *i*, thereby allowing the algorithm to deactivate locations when doing so improves the objective values or reduces constraint violations.

Equations (30)–(31) enforce Eq. (14) by construction and encode heterogeneity through the selected class index $\kappa _i^\star$.

#### (B) Decoding and objective/constraint evaluation

Once a continuous solution *y* is decoded into the binary deployment matrix $\chi$, the resulting configuration is evaluated using the problem objectives and constraints defined in “WSN system model and problem formulation”.

Given *y*, decoding yields $\chi$ via Eqs. (30)–(31). We then evaluate:

1. Coverage: compute $m_{\tau _j}$ using Eq. (16) and $f_{\textrm{cov}}(\chi )$ using Eq. (18).
2. Communication graph: build $\eta ({\bf p}_i,{\bf p}_j)$ using Eqs. (19)–(20) and compute degrees $c_{\textrm{conn},i}$ using Eq. (21).
3. Connectivity: compute $f_{\textrm{conn}}(\chi )$ using Eq. (23).
4. Cost: compute $f_{\textrm{cost}}(\chi )$ using Eq. (24).
5. Constraint violation: detect *K*-coverage and *C*-connectivity violations using
  32 $$\begin{aligned} v(\chi )=\sum _{j=1}^{N_{\textrm{tgt}}}\max \{0,\,K-m_{\tau _j}\} + \sum _{i=1}^{N_{\textrm{loc}}}\max \{0,\,C\cdot a_i-c_{\textrm{conn},i}\}, \end{aligned}$$
  where $$a_i=\sum _{\kappa =1}^{N_{\textrm{cls}}}\chi _i^{\kappa }$$.

The violation function $v(\chi )$ quantifies the degree to which a candidate deployment fails to satisfy the required coverage and connectivity constraints. A value of $v(\chi )=0$ indicates a feasible deployment configuration.

#### (C) Constraint handling mechanism (constraint-domination)

To effectively guide the search toward feasible high-quality solutions, EnMOGKSO adopts a feasibility-first constraint-domination strategy, which is widely used in constrained multi-objective optimization.

EnMOGKSO uses a feasibility-first constraint-domination relation.

For two solutions $\chi ^{(1)}$ and $\chi ^{(2)}$, with violations $v(\chi ^{(1)})$ and $v(\chi ^{(2)})$, the relation is:

33 $$\begin{aligned} \chi ^{(1)} \prec _c \chi ^{(2)}\iff {\left\{ \begin{array}{ll} v(\chi ^{(1)})=0 \ \wedge \ v(\chi ^{(2)})>0, & \text {(feasible preferred)}\\ v(\chi ^{(1)})=v(\chi ^{(2)})=0 \ \wedge \ \chi ^{(1)}\prec \chi ^{(2)}, & \text {(Pareto dominance)}\\ v(\chi ^{(1)})>0 \ \wedge \ v(\chi ^{(2)})>0 \ \wedge \ v(\chi ^{(1)})<v(\chi ^{(2)}), & \text {(smaller violation)}. \end{array}\right. } \end{aligned}$$

This rule prioritizes feasible solutions over infeasible ones and promotes solutions with smaller constraint violations when feasibility has not yet been achieved.

This relation is used in sorting, selection, and archive updates.

Consequently, the external archive maintains only feasible non-dominated solutions, ensuring that the final Pareto front represents practically deployable WSN configurations.

#### (D) Interaction between EnMOGKSO operators and WSN structure

The interaction between the EnMOGKSO search operators and the WSN deployment structure occurs through the encoding–decoding mechanism.

PBA/NBA store continuous vectors *y* but are evaluated after decoding to $\chi (y)$.

This design allows the optimization process to operate in a smooth continuous space while the evaluation phase respects the discrete deployment constraints of the WSN problem.

Levy perturbations diversify *Y* by activating/deactivating sites and switching sensor classes, while NBA-guided refinement biases updates toward feasible deployments with lower cost and stronger coverage/connectivity.

As a result, the algorithm maintains a balance between structural diversity in the deployment configurations and progressive improvement of the multi-objective performance metrics.

### Computational complexity

This subsection analyzes the computational cost of the proposed EnMOGKSO framework with respect to the main problem parameters. Let $N_p$ denote the population size, $N_{\textrm{loc}}$ the number of candidate deployment sites, $N_{\textrm{cls}}$ the number of heterogeneous sensor classes, and $N_{\textrm{tgt}}$ the number of monitoring targets.

During each iteration, every candidate solution must be decoded and evaluated using the coverage, connectivity, and cost objectives. The computational effort is therefore dominated by the objective and constraint evaluation procedures.

Coverage evaluation costs $\mathcal {O}(N_{\textrm{loc}}N_{\textrm{cls}}N_{\textrm{tgt}})$ because the sensing contribution of each sensor class at every deployment location must be assessed with respect to all targets. Connectivity evaluation costs $\mathcal {O}(N_{\textrm{loc}}^2)$ since the communication graph requires pairwise distance checks among active deployment sites.

Considering that these evaluations are performed for each individual in the population, the dominant per-iteration complexity becomes

34 $$\begin{aligned} \mathcal {O}\!\left( N_p\left( N_{\textrm{loc}}\,N_{\textrm{cls}}\,N_{\textrm{tgt}}+N_{\textrm{loc}}^2\right) \right) , \end{aligned}$$

which corresponds to the total cost of evaluating all candidate deployments in one iteration of the algorithm.

In addition to objective evaluation, EnMOGKSO performs archive maintenance operations for storing elite non-dominated solutions. These operations include dominance comparisons and archive updates, which typically incur a computational cost of plus archive maintenance, typically $\mathcal {O}(|\mathcal {A}|N_p)$.

Since the archive size $|\mathcal {A}|$ is bounded in practice, this overhead remains moderate compared with the evaluation cost of the coverage and connectivity objectives. Consequently, the overall computational complexity of EnMOGKSO is primarily governed by the population size and the structural parameters of the WSN deployment problem.

### Architecture of the enhanced GKSO algorithm (EnMOGKSO)

EnMOGKSO extends GKSO with archive-based elitism and hybrid exploration–exploitation:

In the proposed EnMOGKSO framework, the search process is organized through a coordinated balance between exploration and exploitation mechanisms. Global exploration is primarily promoted through Lévy-flight perturbations and stochastic global sampling, which allow agents to escape local optima and explore diverse regions of the decision space.

Conversely, exploitation is guided by elite solutions stored in external archives, namely the Personal Best Archive (PBA) and the Neighborhood Best Archive (NBA). These archives act as leader repositories that provide high-quality search directions for the population. Local refinement operators further intensify the search around promising regions of the Pareto front, thereby improving convergence accuracy.

This explicit separation between exploration-oriented and exploitation-oriented operators follows established principles in swarm intelligence and multi-objective optimization. By maintaining strong exploration capability while preserving elitist guidance through the archives, the algorithm mitigates premature convergence and population stagnation. At the same time, the archive-based leadership mechanism preserves high-quality non-dominated solutions, which helps maintain diversity and ensures steady convergence toward a well-distributed approximation of the Pareto-optimal front.

1. Personal best archive (PBA) and neighborhood best archive (NBA)

To preserve high-quality solutions during the search process, each agent maintains a *Personal Best Archive (PBA)*, which stores the best solution encountered by that agent according to Pareto dominance. Each agent keeps a *Personal Best Archive (PBA)*:

35 $$\begin{aligned} \text {PBA}_i = {\left\{ \begin{array}{ll} X_i^t & \text {if } F(X_i^t) \prec F(\text {PBA}_i) \\ \text {PBA}_i & \text {otherwise} \end{array}\right. } \end{aligned}$$

In addition to individual memory, a global *Neighborhood Best Archive (NBA)* is maintained to store elite non-dominated solutions discovered by the population. This archive provides guidance for the collective search and promotes information sharing among agents.

A global *Neighborhood Best Archive (NBA)* stores elite solutions:

36 $$\begin{aligned} \text {NBA} \leftarrow \text {Update}(\text {NBA} \cup X_i^t), \quad \text {if } F(X_i^t) \prec F(\text {NBA}_{\text {worst}}) \end{aligned}$$

To prevent uncontrolled archive growth and to maintain computational efficiency, the NBA size is restricted by a predefined capacity constraint:

37 $$\begin{aligned} |\text {NBA}| \le N \end{aligned}$$

The combination of PBA and NBA memories provides stronger search guidance compared with traditional single global-best strategies, as it simultaneously preserves individual experience and population-level elite solutions.

2. Advanced search mechanisms

During the hunting stage of the algorithm, Lévy-flight perturbations are introduced to enhance global exploration capability and to reduce the risk of premature convergence. Lévy flights generate occasional long jumps in the search space, enabling agents to escape local optima and investigate unexplored regions.

38 $$\begin{aligned} \text {Levy}(\beta ) = 0.01 \cdot \frac{u}{|v|^{1/\beta }}, \quad u,v \sim \mathcal {N}(0,1) \end{aligned}$$

3. Elite-guided local search strategy

To balance intensification and diversification, EnMOGKSO applies a probabilistic search strategy that alternates between local exploitation and global exploration around the current leader (Silverback).

With probability 60%, local search:

39 $$\begin{aligned} X_i^{\text {local}} = X_{\text {Silverback}} + \mathcal {N}(0,1) \cdot SF + \text {Levy}(\beta ) \end{aligned}$$

With probability 40%, global sampling:

40 $$\begin{aligned} X_i^{\text {global}} = LB + \text {rand} \cdot (UB - LB) \end{aligned}$$

The search scale is controlled by a time-varying scale factor *SF*, which gradually decreases during the optimization process to shift the search behavior from exploration toward exploitation.

41 $$\begin{aligned} SF = \frac{UB - LB}{10} \cdot \left( 1 - \frac{t}{T_{\max }}\right) \end{aligned}$$

After generating candidate solutions, EnMOGKSO performs an elite-guided refinement step using either NBA or PBA information to improve solution quality.

Candidate positions are then refined using NBA or PBA guidance:

- NBA-guided refinement:
  42 $$\begin{aligned} X_i^{t+1} = X_i + \text {rand} \cdot (X_{\text {nbest}} - X_i), \quad X_{\text {nbest}} \in \text {NBA} \end{aligned}$$
- PBA-guided refinement:
  43 $$\begin{aligned} X_i^{t+1} = X_i + \text {rand} \cdot (\text {PBA}_i - X_i) \end{aligned}$$

The refined candidate solutions are evaluated using the multi-objective fitness function, and the better solution is retained for the next iteration. This refinement mechanism enhances exploitation while preserving diversity through multiple elite guidance sources.

### EnMOGKSO workflow for WSN deployment (archive and selection)

EnMOGKSO updates offspring in continuous space *y*, decodes them to $\chi (y)$, and applies selection via $\prec _c$ (Eq. (33)). The external archive $\mathcal {A}$ stores feasible non-dominated deployments and is pruned by crowding when full. Maintaining archive diversity is essential for deployment decision-making because practitioners often require multiple feasible cost-coverage-connectivity trade-offs rather than a single operating point.

## Experimental evaluation of EnMOGKSO

We evaluate EnMOGKSO against MOPSO^59^, NSGA-II^60^, MOEA/D^61^, MOGWO^62^, MOWOA^63^, MOSCA^64^, MOSMA^65^, and IMOMRFO^66^. All comparative methods are executed under equivalent population and iteration budgets with multiple random seeds, and results are reported with dispersion statistics to support reliable comparisons. Benchmarking is performed on the CEC 2020 multi-objective suite and on the defined 3D heterogeneous WSN deployment scenarios in “WSN system model and problem formulation”; no additional external dataset is introduced in this revision. In this revision, we provide mechanism-level interpretation and broad algorithmic comparisons; a full component-wise ablation of all EnMOGKSO operators is explicitly treated as future work (“Conclusion and future work”).

### Evaluation metrics

We use HV, IGD, and PSP as defined in “Performance indicators and metric definitions”. For HV, reference points are fixed to $(1.2,1.2)^T$ (two-objective cases) and $(1.2,1.2,1.2)^T$ (three-objective cases) across all runs.

### Experimental configuration

All runs used the same hardware/software environment (Table 4).

Table 5 lists algorithm settings; shared values are $Num=100$, $MaxIt=500$, and 20 runs.

**Table 4:** Hardware and software configuration used in the experiments.

Configurations	Various components	Characterization
Hardware	CPU	Core (TM) i7
	Frequency	1.70 GHz
	RAM	32 GB
	Hard Drive	500 GB
Software	Operating system	Windows 8
	Language	MATLAB R2013a (8.1.0.604)

**Table 5:** Parameter settings for EnMOGKSO and competing algorithms.

Approaches	Settings configuration
shared configurations	
Agents size: $Num = 100$	
Number of iterations: $MaxIt = 500$	
Independent runs: $R = 20$	
MOGKSO & EnMOGKSO	$archive_{size} = 100$, $h=0.1$
MOEA/D	$archive_{size} = 100$, $T = 0.1$, $\eta = 0.01$, $\delta = 0.9$, $\eta = 30$
MOSMA	$archive_{size} = 100$, $z = 0.03$
NSGA-II	$P_c = 0.8$, $P_m = 0.1$
IMOMRFO	$archive_{size} = 100$, $Coef = \text {CurrentIteration}/\text {MaxIt}$, $S = 2$
MOSCA	$archive_{size} = 100$, $a = 2$, $r_1 = a \cdot \text {CurrentIteration} \cdot (\alpha / \text {MaxIt})$, $r_2 = 6.28 \cdot \text {rand}$, $r_3 = 2 \cdot \text {rand}$, $r_4 = \text {rand}$
MOWOA	$archive_{size} = 100$, $nGrid = 10$, $\beta = 10$, $\alpha = 0.1$, $\gamma = 2$
$MO\_Ring\_PSO\_SCD$	$archive_{size} = 100$, $C_1=2.05, C_2 = 2.05$, $W = 0.7298$
MOGWO	$nGrid = 10$, $\beta = 10$, $\alpha = 0.1$, $\gamma = 2$
MOGTO	$archive_{size} = 100$, $p = 0.03$, $\beta = 3$, $w = 0.8$,
MOPSO	$\phi _1 = 2.05$, $\phi _2 = 2.05$, $\alpha = 0.1$, $\beta = 4$, $nGrid = 10$

Hyperparameters follow source papers with limited preliminary tuning, then remain fixed for all MMF tests.

### CEC 2020 benchmark results

The CEC 2020 multi-objective suite is used to test robustness under diverse front geometries, multimodality, and variable interactions. This helps verify whether algorithmic improvements generalize beyond a single deployment instance and remain stable in irregular, non-convex landscapes.

**Table 6:** Comparative HV results for EnMOGKSO and competing multi-objective algorithms.

Algorithm	MMF1			MMF2			MMF4			MMF5		
	Mean	SD	Rank	Mean	SD	Rank	Mean	SD	Rank	Mean	SD	Rank
EnMOGKSO	0.873	0.000297	1	0.874	0.0000659	1	0.54	0.000356	1	0.873	0.000171	1
MOGKSO	0.872	0.000198	2	0.872	0.000549	2	0.54	0.0000821	2	0.872	0.000153	2
IMOMRFO	0.865	0.00077	7	0.865	0.00108	5	0.53	0.0013	7	0.865	0.00136	7
MOEA/D	0.843	0.00963	10	0.709	0.0793	11	0.513	0.00598	11	0.844	0.00479	9
MOGTO	0.87	0.000292	3	0.843	0.0108	8	0.537	0.000541	3	0.87	0.000381	3
MOGWO	0.842	0.00781	11	0.861	0.11	7	0.522	0.0545	10	0.847	0.096	11
MOPSO	0.869	0.000312	4	0.868	0.000802	3	0.535	0.000367	4	0.869	0.000329	4
MOSCA	0.864	0.00112	8	0.776	0.265	9	0.525	0.00918	8	0.865	0.00127	6
MOSMA	0.868	0.00195	5	0.866	0.000631	4	0.532	0.00145	6	0.864	0.00692	8
MOWOA	0.737	0.0775	12	0.653	0.121	12	0.413	0.0946	12	0.781	0.0214	12
MO_Ring_PSO_SCD	0.867	0.000823	6	0.862	0.00178	6	0.533	0.000893	5	0.868	0.00066	5
NSGA-II	0.844	0.00619	9	0.726	0.0486	10	0.523	0.00466	9	0.84	0.0103	10

Algorithm	MMF7			MMF8			MMF10			MMF11		
	Mean	SD	Rank	Mean	SD	Rank	Mean	SD	Rank	Mean	SD	Rank
EnMOGKSO	0.873	0.000541	1	0.422	0.000263	1	12.9	0.00103	1	14.5	0.00352	1
MOGKSO	0.873	0.000094	2	0.422	0.0000791	2	12.9	0.000355	2	14.5	0.000518	2
IMOMRFO	0.865	0.0014	7	0.411	0.00516	8	12.8	0.012	6	14.5	0.00777	6
MOEA/D	0.854	0.00358	9	0.372	0.0138	10	12.4	0.536	9	14.4	0.0291	8
MOGTO	0.87	0.000383	3	0.419	0.000343	3	12.8	0.0908	8	14.5	0.00468	4
MOGWO	0.833	0.0125	11	0.408	0.575	11	12.8	0.00332	3	14.3	0.021	11
MOPSO	0.868	0.000415	5	0.418	0.000458	4	12.8	0.00396	4	14.5	0.00263	3
MOSCA	0.862	0.00138	8	0.414	0.00149	7	12.8	0.00509	5	14.4	0.0135	7
MOSMA	0.869	0.00147	4	0.415	0.00132	5	12.3	0.204	10	14.4	0.0505	9
MOWOA	0.788	0.0355	12	0.357	0.0986	12	12	0.424	11	13.3	0.63	12
MO_Ring_PSO_SCD	0.865	0.00135	6	0.415	0.00145	6	12.8	0.0242	7	14.5	0.00528	5
NSGA-II	0.849	0.00683	10	0.408	0.00212	9	11.8	0.345	12	14.4	0.0962	10

Algorithm	MMF12			MMF13			MMF14			MMF15		
	Mean	SD	Rank	Mean	SD	Rank	Mean	SD	Rank	Mean	SD	Rank
EnMOGKSO	1.57	0.000153	4	18.4	0.0021	1	2.96	0.246	2	4.24	0.181	2
MOGKSO	1.57	0.000118	1	18.4	0.00225	2	2.86	0.15	4	4.13	0.165	4
IMOMRFO	1.57	0.0034	7	18.4	0.0118	5	2.67	0.282	9	3.9	0.276	8
MOEA/D	1.47	0.125	9	18.2	0.0552	10	2.78	0.168	7	4.12	0.251	5
MOGTO	1.57	0.00013	2	18.4	0.0129	6	2.82	0.102	5	4.19	0.114	3
MOGWO	1.55	0.00329	8	18.2	0.0406	9	1.29	0.0223	12	2.08	0.0237	12
MOPSO	1.57	0.000139	3	18.4	0.00335	3	2.83	0.255	6	4.09	0.159	6
MOSCA	1.57	0.00278	6	18.3	0.0117	8	2.64	0.32	11	3.9	0.301	9
MOSMA	1.41	0.164	11	18.4	0.045	7	3.1	0.351	1	4.4	0.551	1
MOWOA	1.02	0.31	12	17	0.579	12	3	1.31	3	3.69	1.36	11
MO_Ring_PSO_SCD	1.57	0.00122	5	18.4	0.00551	4	2.65	0.237	10	3.95	0.26	7
NSGA-II	1.47	0.154	10	17.9	0.262	11	2.73	0.142	8	3.86	0.405	10

Algorithm	MMF1_e			MMF14_a			MMF15_a			MMF10_I		
	Mean	SD	Rank	Mean	SD	Rank	Mean	SD	Rank	Mean	SD	Rank
EnMOGKSO	0.874	0.000296	1	2.84	0.0854	8	4.24	0.0865	4	12.9	0.00116	1
MOGKSO	0.872	0.000576	2	2.9	0.135	7	4.35	0.252	2	12.9	0.000374	2
IMOMRFO	0.865	0.00253	4	2.81	0.266	9	3.99	0.394	7	12.8	0.0117	7
MOEA/D	0.803	0.1	8	2.93	0.237	6	4.18	0.193	5	12.3	0.564	10
MOGTO	0.87	0.000542	3	3.1	0.204	1	4.37	0.46	3	12.9	0.00388	3
MOGWO	0.844	0.7	9	1.44	0.0432	12	2.43	0.0752	12	12.8	0.00311	4
MOPSO	0.865	0.00648	5	2.95	0.167	4	4.09	0.133	6	12.8	0.00435	5
MOSCA	0.862	0.00676	6	2.98	0.406	3	3.94	0.401	9	12.8	0.00443	6
MOSMA	0.11	0.72	11	2.97	0.254	2	4.37	0.22	1	12.4	0.147	9
MOWOA	0.611	0.27	10	2.83	1.21	10	3.36	1.92	11	12.1	0.388	11
MO_Ring_PSO_SCD	0.858	0.0102	7	2.95	0.304	5	3.95	0.258	8	12.8	0.0171	8
NSGA-II	0.0899	1.51	12	2.48	0.567	11	3.69	0.474	10	11.4	0.117	12

Algorithm	MMF11_I			MMF12_I			MMF13_I			MMF15_I		
	Mean	SD	Rank	Mean	SD	Rank	Mean	SD	Rank	Mean	SD	Rank
EnMOGKSO	14.5	0.00116	1	1.57	0.0000935	1	18.4	0.00374	1	4.22	0.0715	3
MOGKSO	14.5	0.00047	2	1.57	0.0000681	2	18.4	0.00085	2	4.27	0.154	2
IMOMRFO	14.5	0.00768	6	1.57	0.00255	6	18.4	0.00935	6	3.89	0.193	9
MOEA/D	14.4	0.0665	10	1.45	0.143	9	18.2	0.0768	11	4.16	0.138	5
MOGTO	14.5	0.00453	4	1.57	0.00016	4	18.4	0.00717	5	4.19	0.135	4
MOGWO	14.4	0.00764	7	1.55	0.00216	8	18.2	0.0221	10	2.12	0.0261	12
MOPSO	14.5	0.00331	3	1.57	0.000139	3	18.4	0.00352	3	4.08	0.199	6
MOSCA	14.4	0.0153	8	1.57	0.00394	7	18.3	0.0147	8	3.94	0.247	8
MOSMA	14.4	0.0349	9	1.44	0.145	11	18.4	0.0249	7	4.32	0.331	1
MOWOA	13.2	0.636	12	1.1	0.325	12	16.7	1.49	12	3.51	1.65	11
MO_Ring_PSO_SCD	14.5	0.00625	5	1.57	0.00155	5	18.4	0.00472	4	3.84	0.313	10
NSGA-II	14.1	0.105	11	1.45	0.156	10	18.3	0.0937	9	3.99	0.152	7

Algorithm	MMF15_a_I			MMF16_I1			MMF16_I2			MMF16_I3		
	Mean	SD	Rank	Mean	SD	Rank	Mean	SD	Rank	Mean	SD	Rank
EnMOGKSO	4.25	0.06	4	4.24	0.0528	2	4.23	0.063	2	4.27	0.0532	3
MOGKSO	4.34	0.229	2	4.22	0.147	4	4.24	0.167	3	4.22	0.156	4
IMOMRFO	3.96	0.225	8	3.94	0.364	8	4.08	0.27	7	4.03	0.206	7
MOEA/D	4.18	0.227	5	4.22	0.2	5	4.21	0.119	4	4.17	0.234	5
MOGTO	4.36	0.493	3	4.23	0.149	3	4.21	0.126	5	4.28	0.119	2
MOGWO	2.44	0.129	12	2.12	0.0192	12	2.12	0.0152	12	2.18	0.0173	12
MOPSO	4.1	0.194	6	4.07	0.231	6	4.11	0.209	6	4.1	0.258	6
MOSCA	3.98	0.471	9	3.91	0.395	10	3.86	0.421	10	3.9	0.365	9
MOSMA	4.45	0.293	1	4.53	0.532	1	4.39	0.312	1	4.59	0.298	1
MOWOA	3.62	2.17	11	4.04	1.52	9	3.86	1.22	11	3.81	1.78	11
MO_Ring_PSO_SCD	4.01	0.288	7	3.88	0.253	11	3.93	0.151	9	3.93	0.349	8
NSGA-II	3.89	0.349	10	3.97	0.176	7	4.01	0.183	8	3.89	0.288	10

**Table 7:** Comparative IGD results for EnMOGKSO and competing multi-objective algorithms.

Algorithm	MMF1			MMF2			MMF4			MMF5		
	Mean	SD	Rank	Mean	SD	Rank	Mean	SD	Rank	Mean	SD	Rank
EnMOGKSO	0.0444	0.00198	1	0.00941	0.00242	1	0.0285	0.0028	1	0.0779	0.00422	1
MOGKSO	0.08	0.00672	4	0.0395	0.0141	6	0.154	0.0482	8	0.251	0.0493	8
IMOMRFO	0.0834	0.00716	6	0.0165	0.00262	4	0.0611	0.0111	4	0.153	0.0217	4
MOEA/D	0.213	0.03	9	0.271	0.112	9	0.124	0.017	6	0.313	0.0404	9
MOGTO	0.0847	0.0222	5	0.124	0.0354	8	0.125	0.0484	7	0.161	0.0144	6
MOGWO	0.594	0.185	10	0.343	0.218	10	0.509	0.247	10	1.06	0.286	11
MOPSO	0.0636	0.00218	2	0.0113	0.000892	2	0.0423	0.00276	2	0.113	0.00625	2
MOSCA	0.0952	0.00843	7	0.0181	0.00942	5	0.0978	0.0214	5	0.158	0.0173	5
MOSMA	0.136	0.017	8	0.0699	0.0272	7	0.264	0.0608	9	0.213	0.0254	7
MOWOA	0.594	0.185	10	0.343	0.218	10	0.509	0.247	10	1.06	0.286	11
MO_Ring_PSO_SCD	0.0734	0.00716	3	0.0158	0.00239	3	0.0454	0.00313	3	0.122	0.00621	3
NSGA-II	0.852	0.00378	12	0.768	0.0604	12	0.523	0.00228	12	0.853	0.0063	10

Algorithm	MMF7			MMF8			MMF10			MMF11		
	Mean	SD	Rank	Mean	SD	Rank	Mean	SD	Rank	Mean	SD	Rank
EnMOGKSO	0.0245	0.002	1	0.0727	0.0224	1	0.00171	0.000131	1	0.00368	0.000203	2
MOGKSO	0.0799	0.0179	6	0.665	0.166	7	0.00188	0.0000539	2	0.00325	0.000137	1
IMOMRFO	0.069	0.00855	5	0.254	0.123	5	0.0047	0.000252	4	0.00808	0.000437	6
MOEA/D	0.104	0.0223	8	1.13	0.514	8	0.149	0.146	11	0.0202	0.00732	9
MOGTO	0.0546	0.0142	4	1.87	1.05	12	0.00787	0.0145	7	0.0054	0.000763	3
MOGWO	0.364	0.111	10	1.44	0.686	10	0.0588	0.0518	9	0.0957	0.0666	10
MOPSO	0.0357	0.00266	2	0.0819	0.00609	2	0.00295	0.0000947	3	0.00646	0.000293	4
MOSCA	0.0801	0.00959	7	0.211	0.0825	4	0.00536	0.000645	5	0.0106	0.000938	7
MOSMA	0.142	0.0362	9	1.34	0.588	9	0.0345	0.0397	8	0.0146	0.00525	8
MOWOA	0.364	0.111	10	1.44	0.686	10	0.0588	0.0518	9	0.0957	0.0666	10
MO_Ring_PSO_SCD	0.0423	0.00308	3	0.0993	0.0121	3	0.00628	0.000804	6	0.0077	0.000785	5
NSGA-II	0.854	0.00285	12	0.415	0.000981	6	12.8	0.0151	12	14.4	0.0291	12

Algorithm	MMF12			MMF13			MMF14			MMF15		
	Mean.	SD .	Rank	Mean.	SD .	Rank	Mean.	SD .	Rank	Mean.	SD .	Rank
EnMOGKSO	0.00156	0.000128	2	0.0436	0.00482	3	0.0701	0.00682	1	0.05	0.00397	1
MOGKSO	0.0015	0.00011	1	0.104	0.0332	7	0.108	0.0178	5	0.0576	0.00609	2
IMOMRFO	0.00333	0.00031	5	0.0447	0.00404	3	0.104	0.0092	4	0.0735	0.0049	5
MOEA/D	0.0166	0.00885	9	0.0843	0.0202	5	0.255	0.0534	8	0.0851	0.00634	8
MOGTO	0.00218	0.000428	3	0.155	0.0441	8	0.281	0.0116	9	0.0663	0.0078	3
MOGWO	0.0895	0.0712	10	0.212	0.0497	9	0.371	0.141	10	0.293	0.114	10
MOPSO	0.00282	0.000146	4	0.0385	0.00115	1	0.0949	0.00565	2	0.0703	0.00307	4
MOSCA	0.0035	0.000332	6	0.059	0.00966	4	0.127	0.00851	7	0.0983	0.0081	9
MOSMA	0.00878	0.00253	8	0.087	0.0153	6	0.116	0.0144	6	0.0825	0.0116	7
MOWOA	0.0895	0.0712	10	0.212	0.0497	9	0.371	0.141	10	0.293	0.114	10
MO_Ring_PSO_SCD	0.00352	0.00026	7	0.0413	0.00204	2	0.0994	0.0052	3	0.0744	0.00405	6
NSGA-II	1.52	0.0448	12	18.2	0.0435	11	2.68	0.142	12	3.77	0.294	12

Algorithm	MMF1_e			MMF14_a			MMF15_a			MMF10_I		
	Mean	SD	Rank	Mean	SD	Rank	Mean	SD	Rank	Mean	SD	Rank
EnMOGKSO	0.195	0.0444	1	0.0809	0.00318	1	0.0556	0.00238	1	0.0201	0.0000643	1
MOGKSO	1.17	0.358	6	0.122	0.0226	5	0.0691	0.00836	2	0.0201	0.000026	2
IMOMRFO	3.19	0.465	9	0.115	0.00588	4	0.0817	0.00503	5	0.163	0.00206	4
MOEA/D	3.3	0.844	10	0.233	0.0559	7	0.107	0.00933	6	0.228	0.0225	11
MOGTO	3.05	0.682	8	0.289	0.0981	8	0.159	0.034	8	0.202	0.000401	9
MOGWO	3.57	1.27	11	0.391	0.157	9	0.308	0.17	9	0.198	0.0481	7
MOPSO	0.2	0.0424	2	0.102	0.0043	2	0.0792	0.005	3	0.186	0.0193	6
MOSCA	1.5	0.911	7	0.573	0.00555	11	0.334	0.00612	11	0.203	0.000272	10
MOSMA	1.09	0.447	5	0.231	0.0544	6	0.136	0.0125	7	0.0615	0.0281	3
MOWOA	3.57	1.27	11	0.391	0.157	9	0.308	0.17	9	0.198	0.0481	7
MO_Ring_PSO_SCD	0.299	0.0667	3	0.109	0.00665	3	0.0801	0.00526	4	0.164	0.00622	5
NSGA-II	0.884	0.027	4	2.98	0.268	12	4.13	0.401	12	12.8	0.0443	12

Algorithm	MMF11_I			MMF12_I			MMF13_I			MMF15_I		
	Mean	SD	Rank	Mean	SD	Rank	Mean	SD	Rank	Mean	SD	Rank
EnMOGKSO	0.249	0.000484	6	0.245	0.000347	6	0.269	0.003	4	0.243	0.0239	4
MOGKSO	0.249	0.000312	6	0.245	0.000372	6	0.334	0.0212	8	0.243	0.0233	4
IMOMRFO	0.233	0.0223	2	0.198	0.0438	1	0.265	0.0119	3	0.205	0.0196	3
MOEA/D	0.259	0.00354	9	0.253	0.00765	9	0.31	0.0319	7	0.301	0.00424	9
MOGTO	0.253	0.000956	8	0.247	0.000445	8	0.376	0.032	9	0.286	0.00559	8
MOGWO	0.292	0.0552	10	0.299	0.113	10	0.394	0.0539	10	0.406	0.166	10
MOPSO	0.246	0.0134	4	0.244	0.000432	5	0.264	0.00746	2	0.174	0.0148	1
MOSCA	0.246	0.0135	5	0.243	0.00307	4	0.286	0.0122	5	0.243	0.024	4
MOSMA	0.244	0.0226	3	0.221	0.0342	3	0.291	0.0199	6	0.273	0.0189	7
MOWOA	0.292	0.0552	10	0.299	0.113	10	0.394	0.0539	10	0.406	0.166	10
MO_Ring_PSO_SCD	0.216	0.0272	1	0.207	0.0441	2	0.246	0.0135	1	0.178	0.0182	2
NSGA-II	14.5	0.00489	12	1.57	0.000742	12	18.4	0.00667	12	4.27	0.372	12

Algorithm	MMF15_a_I			MMF16_l1			MMF16_l2			MMF16_l3		
	Mean	SD	Rank	Mean	SD	Rank	Mean	SD	Rank	Mean	SD	Rank
EnMOGKSO	0.215	0.0082	4	0.164	0.00772	3	0.295	0.0246	6	0.212	0.00992	4
MOGKSO	0.225	0.00835	5	0.184	0.00727	5	0.294	0.0254	5	0.229	0.015	5
IMOMRFO	0.204	0.0133	3	0.164	0.018	3	0.245	0.0335	3	0.2	0.0182	3
MOEA/D	0.261	0.00501	6	0.256	0.0269	8	0.369	0.00506	9	0.309	0.0381	8
MOGTO	0.289	0.052	8	0.269	0.0429	9	0.361	0.00541	8	0.315	0.0586	9
MOGWO	0.365	0.215	9	0.356	0.139	10	0.43	0.0805	10	0.343	0.183	10
MOPSO	0.174	0.0105	1	0.147	0.00774	1	0.204	0.0164	1	0.175	0.00991	1
MOSCA	0.574	0.0033	11	0.188	0.0127	6	0.277	0.0273	4	0.232	0.0155	6
MOSMA	0.273	0.0166	7	0.203	0.0138	7	0.325	0.0325	7	0.252	0.0189	7
MOWOA	0.365	0.215	9	0.356	0.139	10	0.43	0.0805	10	0.343	0.183	10
MO_Ring_PSO_SCD	0.19	0.0116	2	0.156	0.00762	2	0.217	0.0173	2	0.183	0.00907	2
NSGA-II	4.2	0.41	12	3.99	0.228	12	4.08	0.206	12	4.15	0.147	12

**Table 8:** Comparative PSP results for EnMOGKSO and competing multi-objective algorithms.

Algorithm	MMF1			MMF2			MMF4			MMF5		
	Mean	SD	Rank	Mean	SD	Rank	Mean	SD	Rank	Mean	SD	Rank
EnMOGKSO	22.5	0.978	1	113	27.5	1	35.3	3.3	1	12.8	0.675	1
MOGKSO	12.3	1.1	4	28	8.81	6	6.78	2.55	8	3.76	0.904	8
IMOMRFO	11.9	0.979	6	61.2	9.47	4	16.5	2.84	4	6.52	0.845	4
MOEA/D	4.39	0.666	9	3.26	2.35	11	7.94	1.18	7	2.89	0.482	9
MOGTO	12.2	2.39	5	8.67	2.35	9	9.17	3.67	6	6.19	0.579	6
MOGWO	2.81	0.868	11	8.86	9	8	1.03	0.125	12	0.78	0.167	11
MOPSO	15.6	0.55	2	87.8	7.17	2	23.5	1.44	2	8.81	0.479	2
MOSCA	10.5	0.915	7	48	20.1	5	10.4	1.99	5	6.33	0.701	5
MOSMA	7.32	1.01	8	16.8	7.21	7	4.13	2.29	9	4.63	0.567	7
MOWOA	1.25	0.672	12	3	1.42	12	2.31	2.63	11	0.477	0.295	12
MO_Ring_PSO_SCD	13.5	0.62	3	63	9.09	3	21.7	1.45	3	8.13	0.414	3
NSGA-II	3.78	0.598	10	3.59	1.59	10	3.81	0.964	10	2.46	0.367	10

Algorithm	MMF7			MMF8			MMF10			MMF11		
	Mean	SD	Rank	Mean	SD	Rank	Mean	SD	Rank	Mean	SD	Rank
EnMOGKSO	40.6	3.13	1	14.4	3.34	1	589	46.8	1	308	12.9	1
MOGKSO	12.2	2.74	5	1.16	0.309	6	533	15.4	2	273	14.9	2
IMOMRFO	12	2.51	6	4.23	1.56	5	212	11.4	4	123	6.59	6
MOEA/D	7.73	2.16	8	0.877	0.492	7	11.3	8.31	11	52.7	20.4	10
MOGTO	19	4.86	4	0.579	0.572	11	212	57.5	4	189	28.1	3
MOGWO	0.21	0.12	12	0.106	0.0853	12	85.3	21	8	48.7	25.5	11
MOPSO	27.9	2.11	2	12.1	0.885	2	339	10.6	3	155	6.95	4
MOSCA	11.3	2.24	7	5.18	1.71	4	189	20.5	6	95.1	8.01	7
MOSMA	6.65	2.5	9	0.856	0.606	8	65.8	48.8	9	76.1	23.2	9
MOWOA	0.633	0.43	11	0.606	0.353	10	23.1	9.29	10	13.4	8.13	12
MO_Ring_PSO_SCD	23.3	1.66	3	10.1	1.17	3	160	19	7	131	12.9	5
NSGA-II	5.19	0.795	10	0.659	0.169	9	4.7	2.5	12	85.1	15.9	8

Algorithm	MMF12			MMF13			MMF14			MMF15		
	Mean	SD	Rank	Mean	SD	Rank	Mean	SD	Rank	Mean	SD	Rank
EnMOGKSO	644	49	2	23	2.43	3	14.4	1.2	1	20.1	1.43	1
MOGKSO	669	46.4	1	9.23	4.2	8	9.52	1.46	5	17.5	1.65	2
IMOMRFO	303	26.7	5	22	1.94	4	9.63	0.829	4	13.6	0.917	5
MOEA/D	73	32	10	11.4	2.65	7	2.8	2.14	9	11.6	1	8
MOGTO	475	84.1	3	1.99	1.65	11	1.59	1.02	11	15.3	1.73	3
MOGWO	197	82.1	8	1.01	0.512	12	0.137	0.0873	12	0.733	0.182	12
MOPSO	355	17.9	4	25.8	0.738	1	10.6	0.603	2	14.2	0.634	4
MOSCA	288	28.5	6	16.8	2.82	5	7.92	0.519	7	10.1	0.856	9
MOSMA	121	26.3	9	11.8	2	6	8.71	1.04	6	12.3	1.68	7
MOWOA	17.3	12	12	3.01	1.93	10	2.67	1.8	10	3.5	2.62	11
MO_Ring_PSO_SCD	286	20.9	7	24.1	1.17	2	10.1	0.513	3	13.5	0.71	6
NSGA-II	63	39	11	6.09	1.57	9	5.46	0.541	8	6.67	1.19	10

Algorithm	MMF1_e			MMF14_a			MMF15_a			MMF10_I		
	Mean	SD	Rank	Mean	SD	Rank	Mean	SD	Rank	Mean	SD	Rank
EnMOGKSO	5.3	1.13	1	12.4	0.48	1	18	0.76	1	0.133	0.02	11
MOGKSO	0.599	0.214	6	8.44	1.57	5	14.6	1.51	2	0.156	0.00698	10
IMOMRFO	0.107	0.0738	10	8.6	0.44	4	12.2	0.731	5	6.06	0.262	2
MOEA/D	0.0874	0.129	11	4.09	1.06	8	9.32	0.841	6	0.359	0.496	7
MOGTO	0.147	0.182	9	3.86	1.37	9	6.55	1.35	8	0.0925	0.0404	12
MOGWO	0.0858	0.106	12	0.691	0.509	11	1.32	0.806	11	0.192	0.0295	9
MOPSO	5.04	0.698	2	9.73	0.396	2	12.6	0.76	3	2.6	2.88	6
MOSCA	0.752	0.654	5	0.0328	0.147	12	0	0	12	0.23	0.0174	8
MOSMA	0.958	0.394	4	4.56	1.17	6	7.44	0.731	7	18.7	6.7	1
MOWOA	0.173	0.332	8	2.19	1.61	10	1.86	1.95	10	3.34	3.23	5
MO_Ring_PSO_SCD MO_Ring_PSO_SCD	3.31	0.761	3	9.12	0.52	3	12.5	0.8	4	6.04	0.195	3
NSGA-II	0.47	0.128	7	4.16	0.219	7	5.1	0.881	9	5.27	2.25	4

Algorithm	MMF11_I			MMF12_I			MMF13_I			MMF15_I		
	Mean	SD	Rank	Mean	SD	Rank	Mean	SD	Rank	Mean	SD	Rank
EnMOGKSO	0.552	0.0483	8	0.465	0.0358	9	1.74	0.0319	5	3.35	1.32	7
MOGKSO	0.526	0.0385	9	0.438	0.0525	10	1.13	0.294	9	3.66	1.06	6
IMOMRFO	2.09	1.65	3	3.61	2.67	1	2.12	0.543	2	4.88	0.476	3
MOEA/D	0.213	0.0693	12	0.717	0.289	7	1.33	0.315	8	0.61	0.0968	11
MOGTO	0.259	0.197	11	0.283	0.0803	12	0.426	0.409	11	1.17	0.362	10
MOGWO	0.327	0.154	10	0.413	0.142	11	0.206	0.0787	12	0.128	0.0705	12
MOPSO	1.01	1.01	7	0.614	0.047	8	1.86	0.278	3	5.77	0.518	1
MOSCA	1.11	0.977	5	0.748	0.273	6	1.63	0.216	7	3.88	0.749	5
MOSMA	1.19	1.15	4	2.32	2.28	3	1.69	0.12	6	2.4	0.976	8
MOWOA	1.1	1.17	6	1.83	2.88	4	0.79	0.363	10	1.55	1.53	9
MO_Ring_PSO_SCD	3.32	1.99	1	2.96	2.67	2	2.68	0.659	1	5.67	0.554	2
NSGA-II	2.15	1.51	2	0.847	0.275	5	1.76	0.242	4	3.94	0.458	4

Algorithm	MMF15_a_I			MMF16_l1			MMF16_l2			MMF16_l3		
	Mean	SD	Rank	Mean	SD	Rank	Mean	SD	Rank	Mean	SD	Rank
EnMOGKSO	4	0.512	4	5.41	0.949	4	2.88	0.63	7	3.9	0.54	6
MOGKSO	3.71	0.359	6	5.15	0.64	6	3.09	0.61	6	3.83	0.727	7
IMOMRFO	4.69	0.467	3	6.13	0.717	3	4.01	0.713	3	4.86	0.613	3
MOEA/D	3.01	0.0614	8	1.48	1.01	10	0.611	0.0582	11	1.52	0.835	10
MOGTO	3.11	0.741	7	1.41	0.89	11	0.867	0.23	10	1.03	0.34	11
MOGWO	0.85	0.602	11	0.103	0.0343	12	0.115	0.0536	12	0.0842	0.0562	12
MOPSO	5.72	0.352	1	6.8	0.355	1	4.93	0.417	1	5.73	0.313	1
MOSCA	0	0	12	5.28	0.382	5	3.33	0.593	5	4	0.471	4
MOSMA	3	0.255	9	4	0.738	8	1.94	0.804	8	3.09	0.635	8
MOWOA	1.34	1.12	10	2.12	1.58	9	1.62	0.828	9	2.31	1.72	9
MO_Ring_PSO_SCD	5.14	0.415	2	6.42	0.306	2	4.63	0.351	2	5.44	0.312	2
NSGA-II	3.79	0.594	5	4.6	0.293	7	3.77	0.499	4	3.97	0.278	5

Tables 6, 7, 8 show that EnMOGKSO is the most consistent high-performing method under the same budget, with top/near-top ranks and generally lower dispersion .

**Fig. 1:**
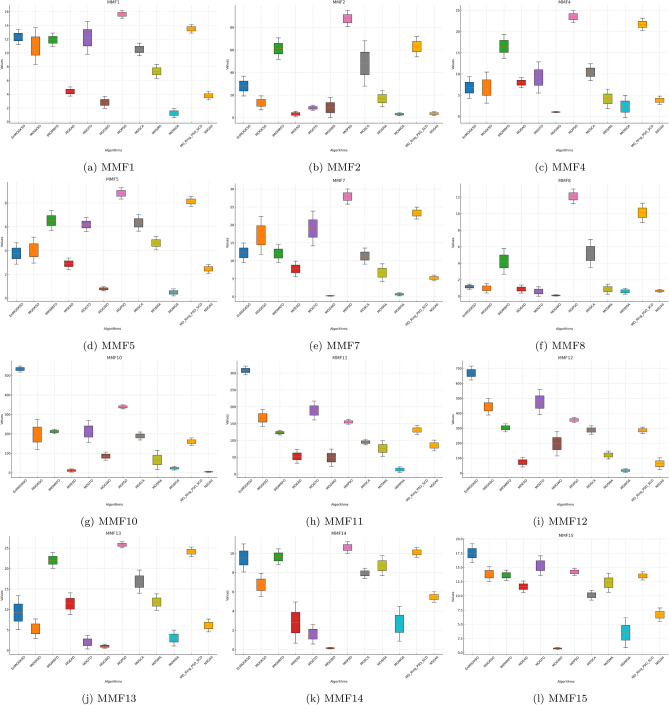
HV boxplots for MMF1 to MMF15 functions.

**Fig. 2:**
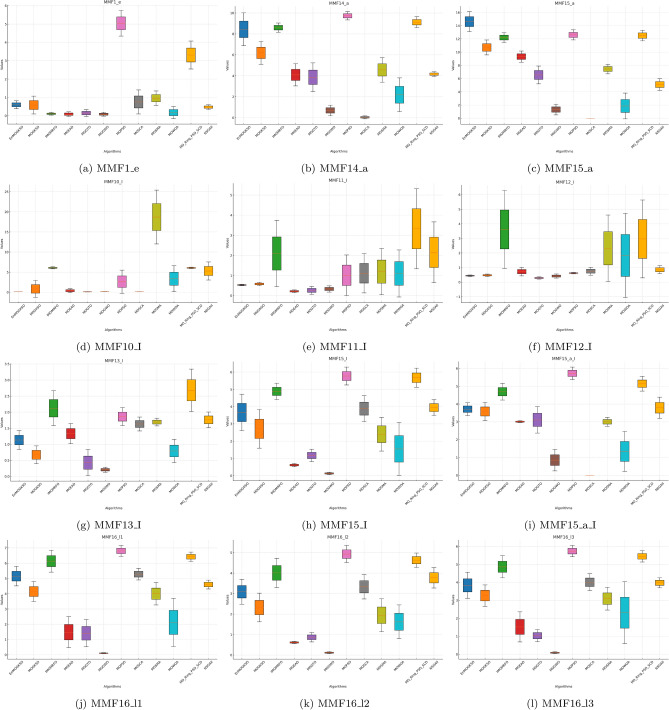
HV boxplots for MMF1_e to MMF16_l3 functions.

**Fig. 3:**
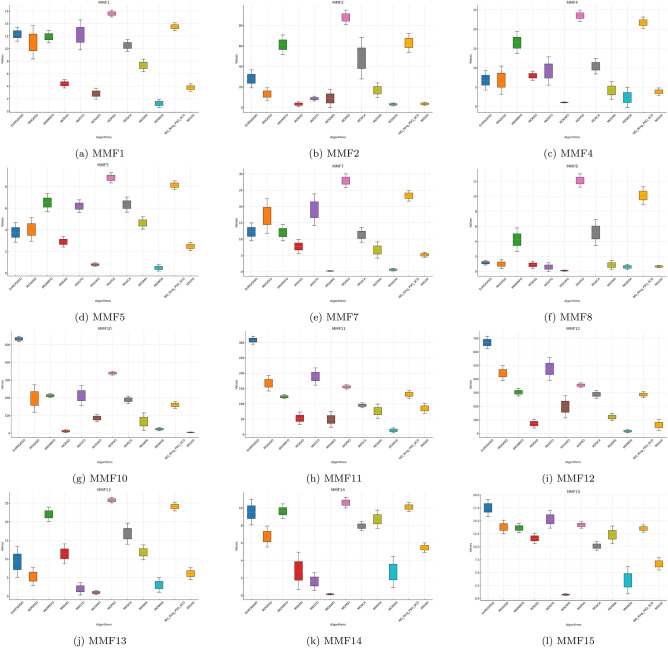
PSP boxplots for MMF1 to MMF15 functions across algorithms.

**Fig. 4:**
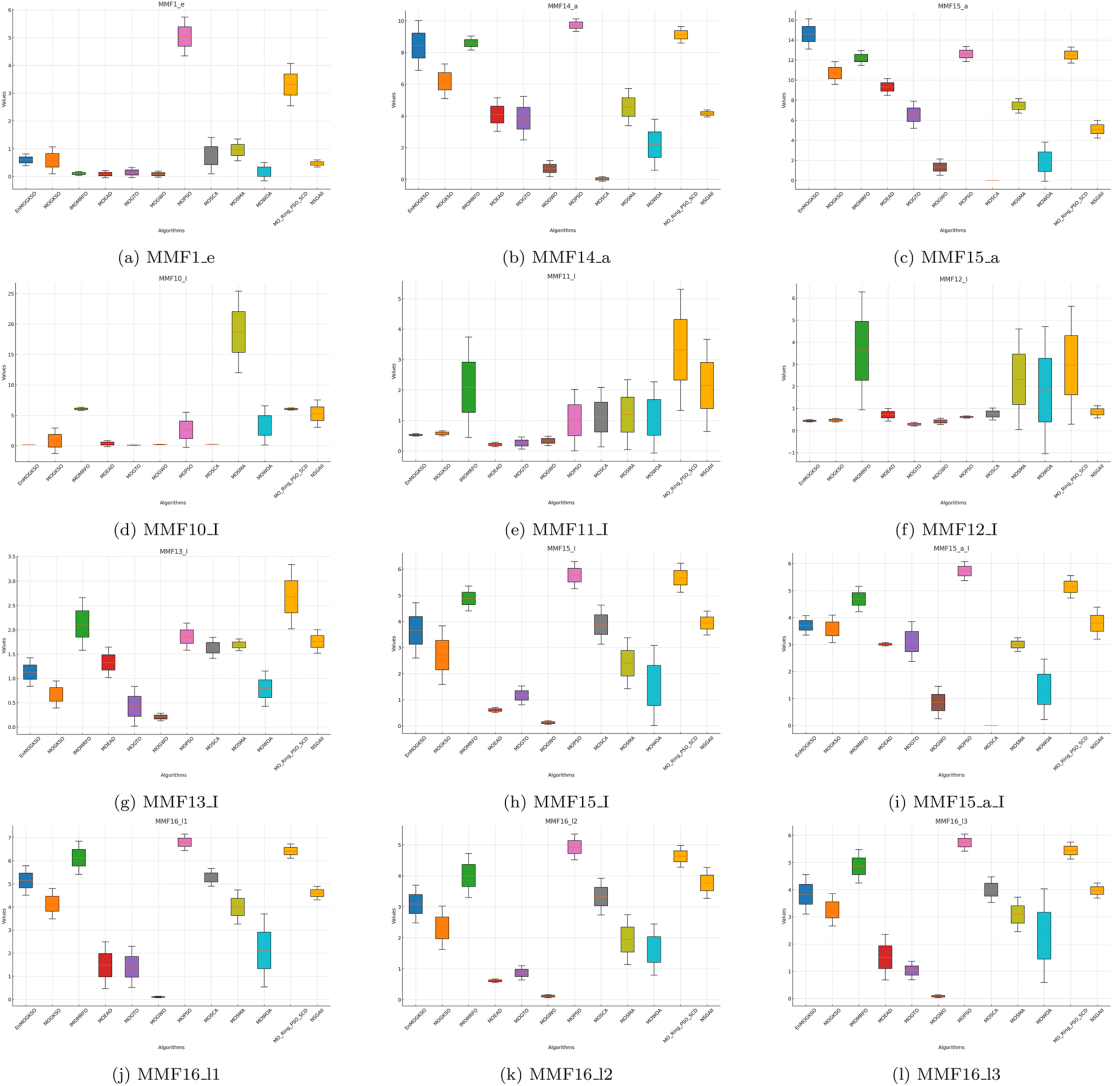
PSP boxplots for MMF1_e to MMF16_l3 functions across algorithms.

**Fig. 5:**
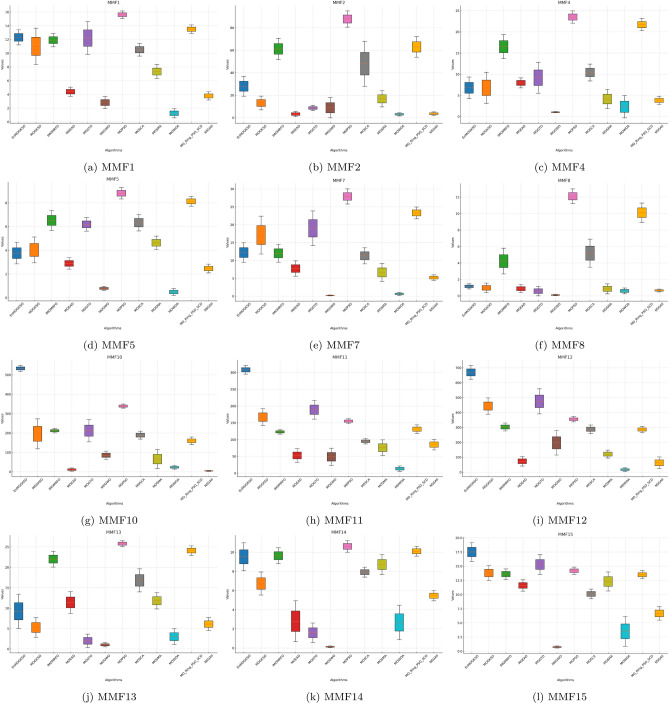
IGD boxplots for MMF1 to MMF15 functions across algorithms.

**Fig. 6:**
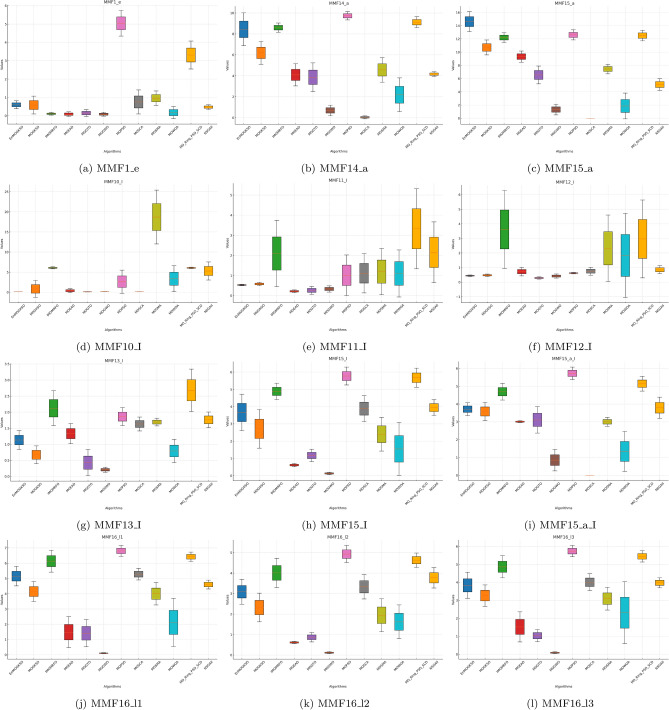
IGD boxplots for MMF1_e to MMF16_l3 functions across algorithms.

The boxplots in Figs. 1, 2, 3, 4, 5, 6 are consistent with the tabulated results: EnMOGKSO maintains high HV, low IGD, and competitive PSP, with tighter spread on most functions, indicating stronger run-to-run stability.

**Fig. 7:**
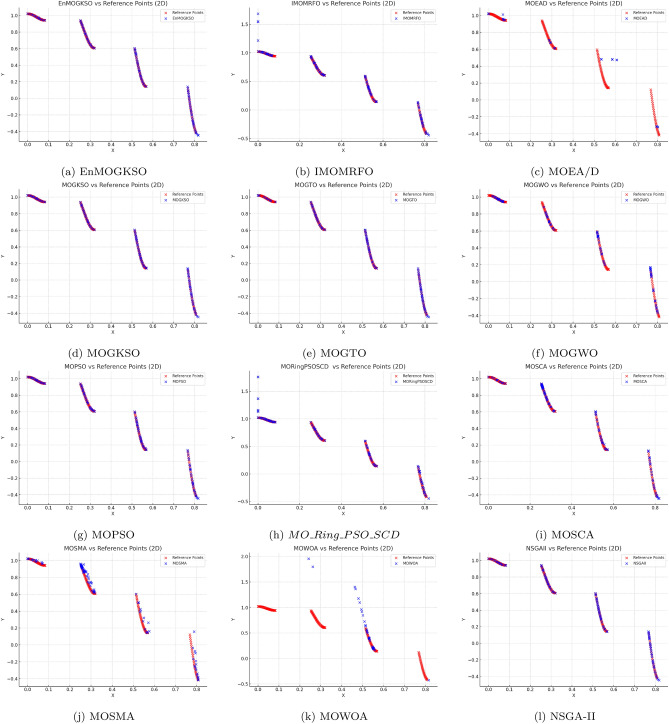
PF comparison against reference points on $MMF10-I$ across algorithms.

**Fig. 8:**
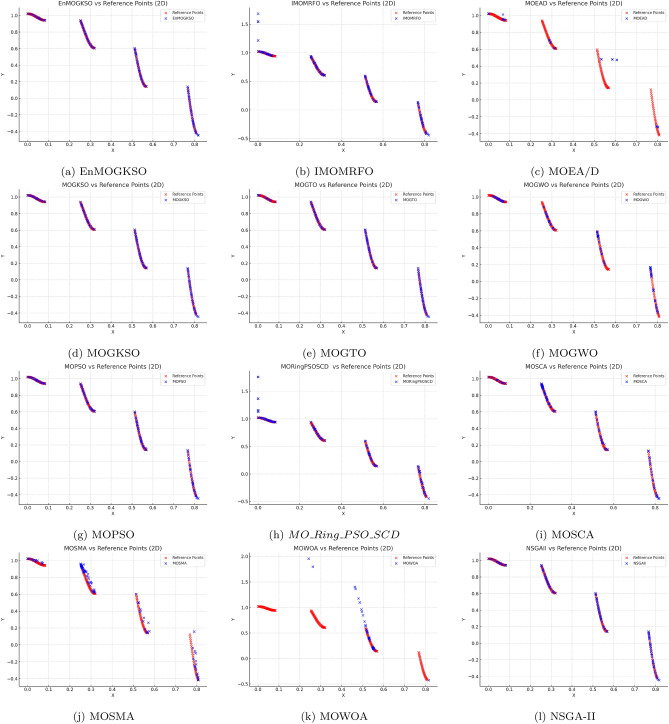
PF comparison against reference points on *MMF*12 across algorithms.

**Fig. 9:**
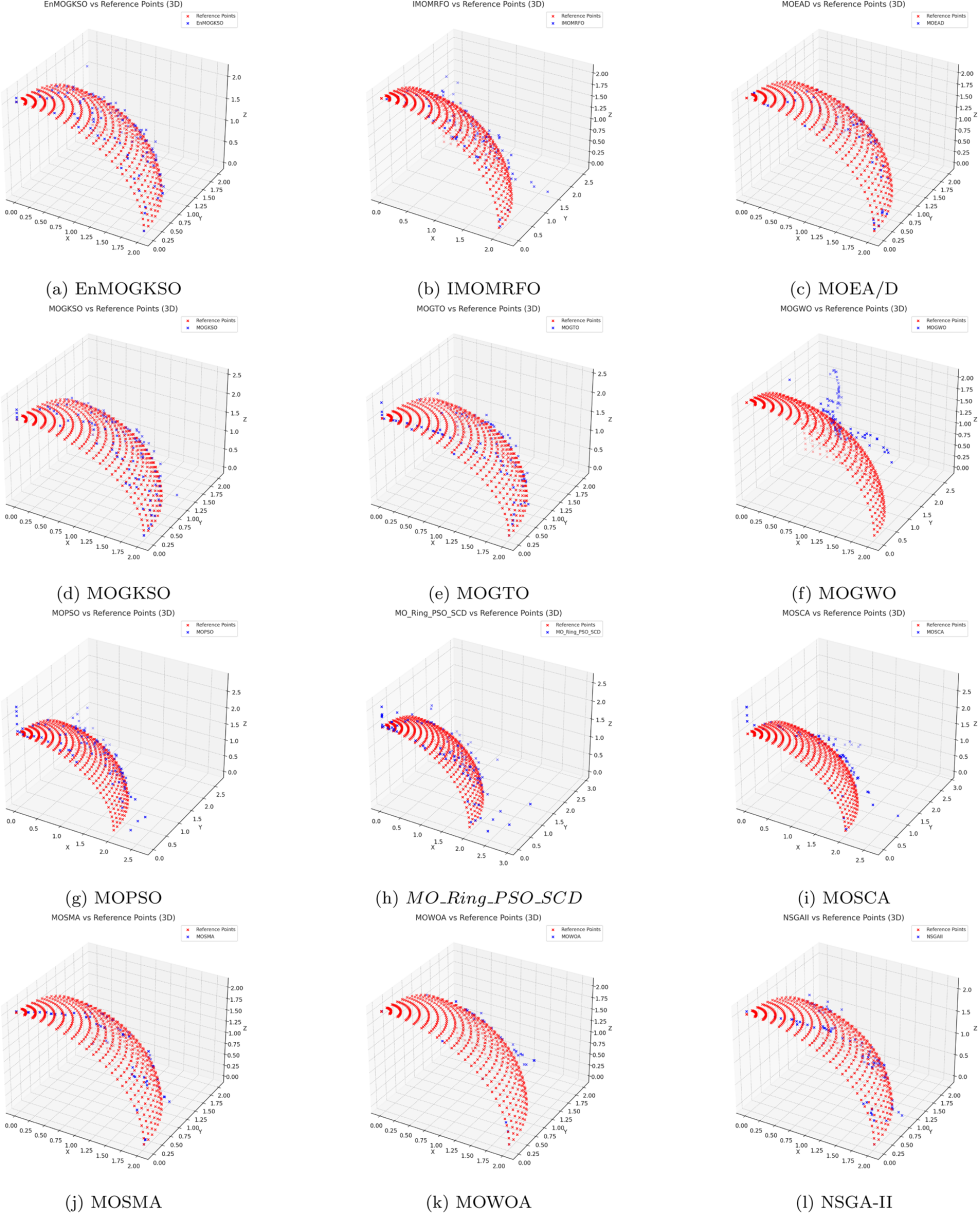
PF comparison against reference points on $MMF15_a$ across algorithms.

**Fig. 10:**
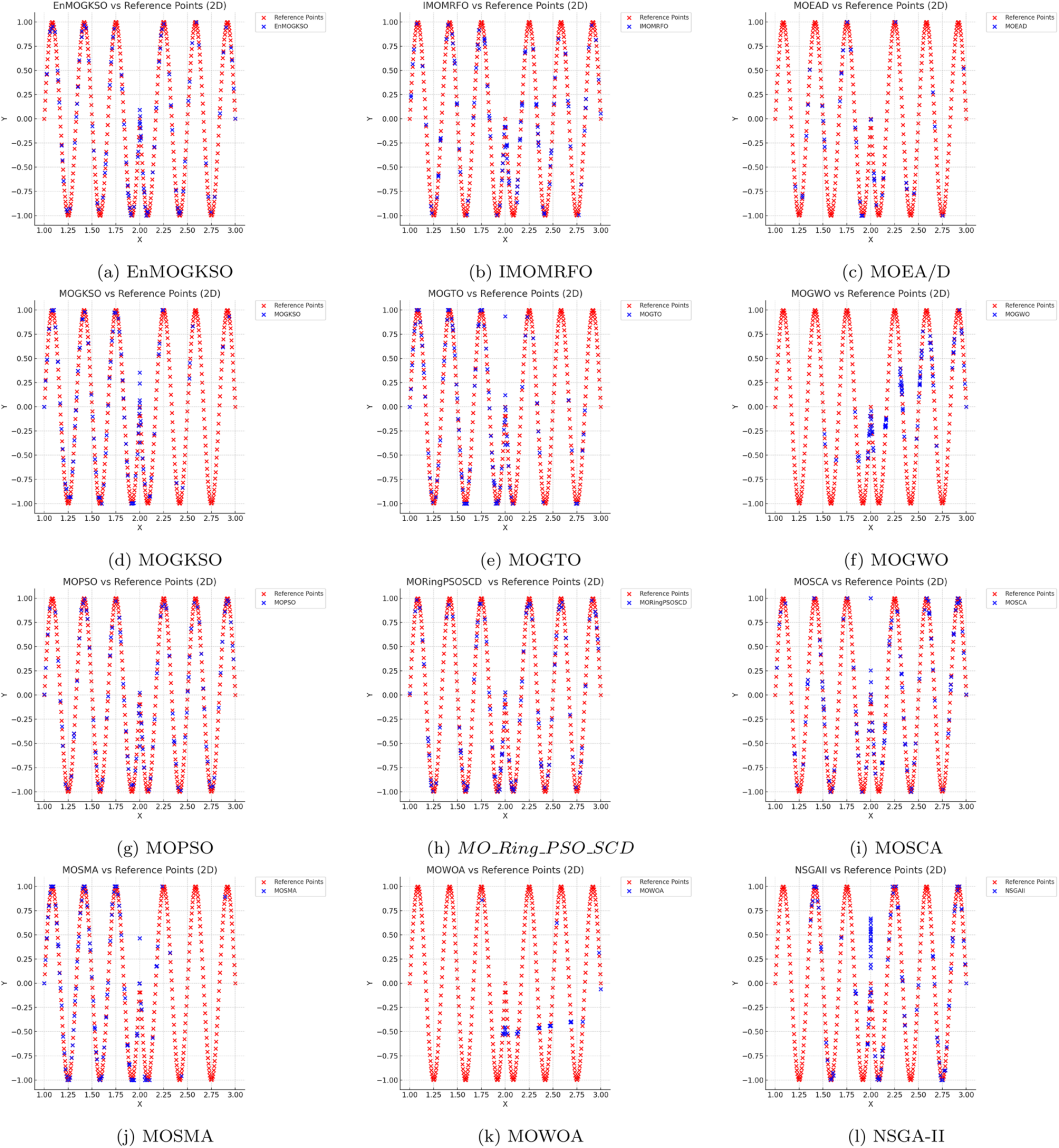
PS comparison against reference points on MMF1 across algorithms.

**Fig. 11:**
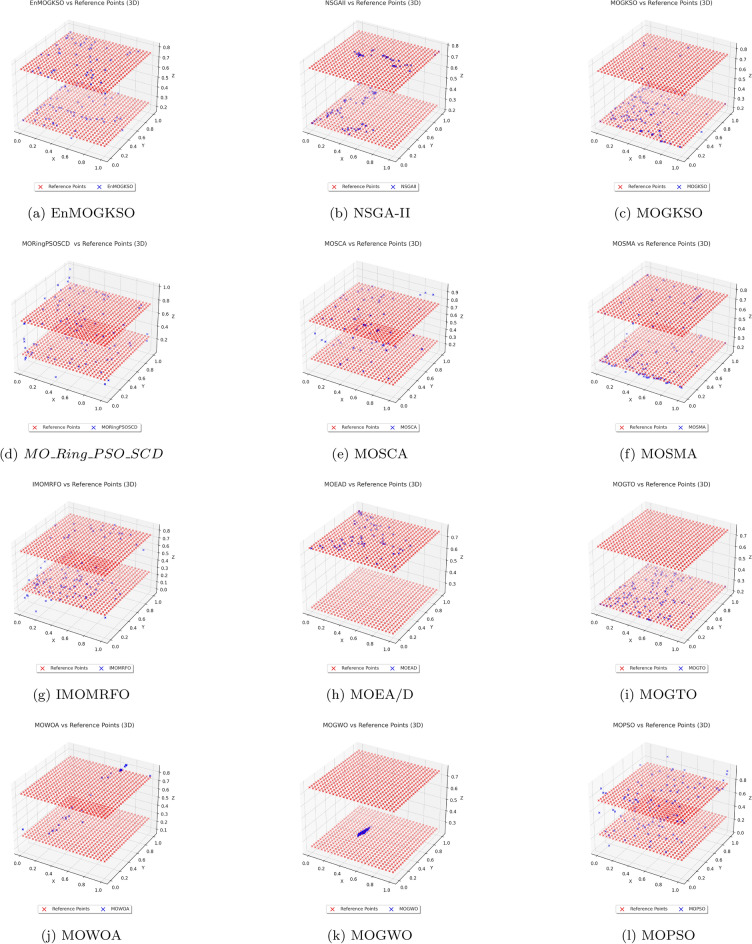
PF comparison against reference points on MMF14 across algorithms.

**Fig. 12:**
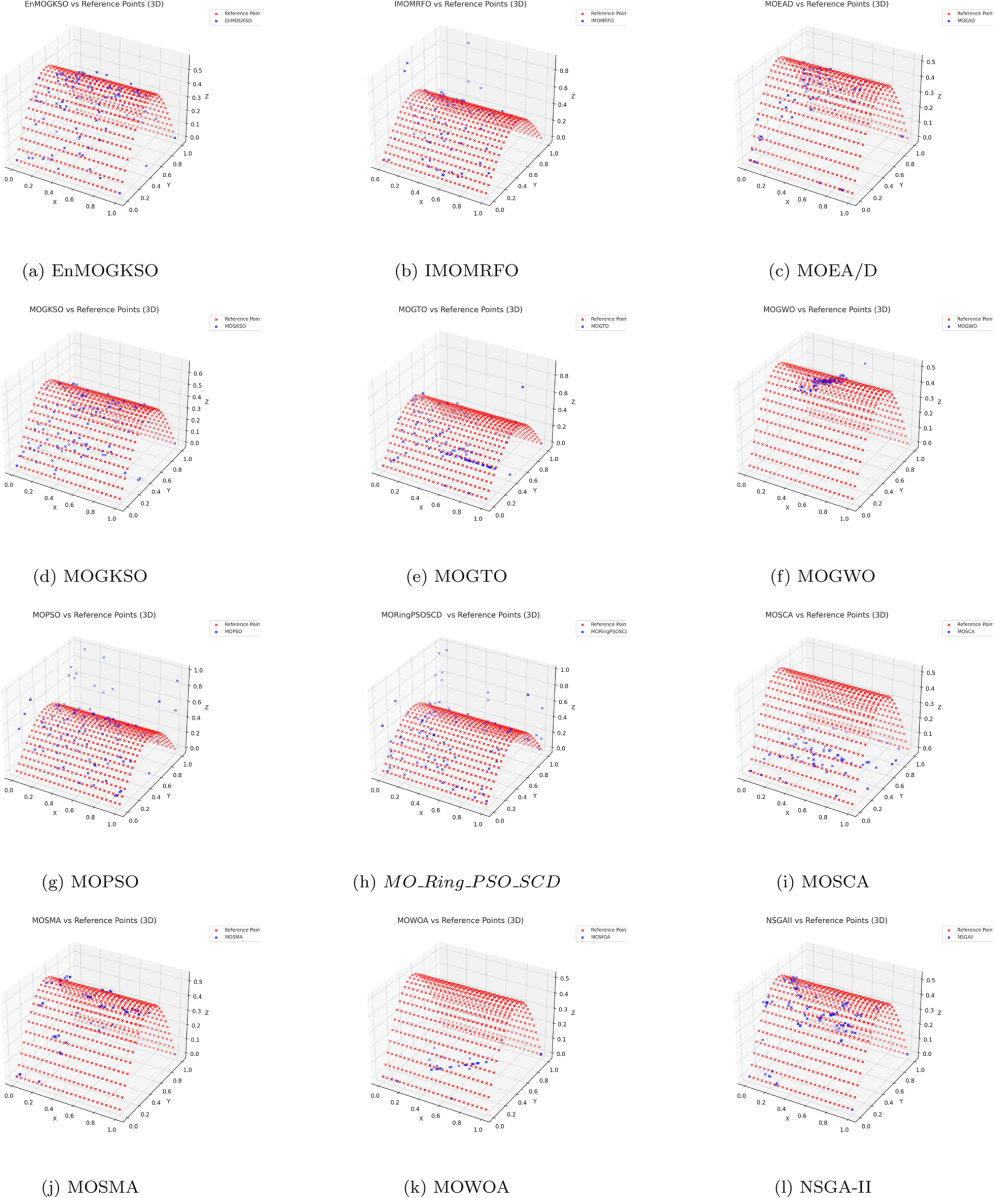
PS comparison against reference points on $MMF15_a$ across algorithms.

Representative PF/PS visualizations (Figs. 7, 8, 9, 10, 11 and 12) show more uniform front tracking for EnMOGKSO, whereas competitors are less consistent on disconnected or multimodal cases.

Pairwise tests align with the metric tables: EnMOGKSO is significant against most competitors, with only a few non-significant comparisons.

### Statistical Significance Analysis

Table 9 ranks EnMOGKSO first in HV (2.04) and IGD (2.38), and third in PSP (3.29).

**Table 9:** Scores and Friedman mean ranks for compared algorithms based on HV, IGD, and PSP.

Algorithms	EnMOGKSO	MOGKSO	IMOMRFO	MOEA/D	MOGTO	MOGWO	MOPSO	MOSCA	MOSMA	MOWOA	MO_Ring_PSO_SCD	NSGA-II
Scores of HV	2.04	2.46	6.92	7.96	3.83	9.92	4.54	7.71	5.25	10.96	6.63	9.79
Ranking	1	2	7	9	3	11	4	8	5	12	6	10
Scores of IGD	2.38	4.83	4.08	8.25	7.29	9.75	2.46	6.46	6.67	9.75	3.17	11.29
Ranking	1	5	4	9	8	10	2	6	7	11	3	12
Scores of PSP	3.29	5.79	4.13	8.96	8.21	11	2.75	6.63	6.92	9.67	3.13	7.5
Ranking	3	5	4	10	9	12	1	6	7	11	2	8

**Table 10:** Wilcoxon signed-rank test results for HV, IGD, and PSP when comparing EnMOGKSO with competing algorithms ($p \le 0.05$ indicates significance).

Algorithm	HV	IGD	PSP
EnMOGKSO vs. MOPSO	$9.93 \times 10^{-5}\ (\text {S})$	$3.96 \times 10^{-1}\ (\text {NS})$	$9.03 \times 10^{-3}\ (\text {S})$
EnMOGKSO vs. MOGWO	$9.3 \times 10^{-6}\ (\text {S})$	$9.4 \times 10^{-6}\ (\text {S})$	$1.21 \times 10^{-5}\ (\text {S})$
EnMOGKSO vs. MOWOA	$2.27 \times 10^{-5}\ (\text {S})$	$1.07 \times 10^{-5}\ (\text {S})$	$4.67 \times 10^{-5}\ (\text {S})$
EnMOGKSO vs. MOSCA	$1.33 \times 10^{-4}\ (\text {S})$	$1.13 \times 10^{-4}\ (\text {S})$	$2.80 \times 10^{-4}\ (\text {S})$
EnMOGKSO vs. MOSMA	$4.71 \times 10^{-1}\ (\text {NS})$	$2.03 \times 10^{-4}\ (\text {S})$	$2.52 \times 10^{-4}\ (\text {S})$
EnMOGKSO vs. MO_Ring_PSO_SCD	$5.22 \times 10^{-4}\ (\text {S})$	$4.57 \times 10^{-1}\ (\text {NS})$	$7.14 \times 10^{-4}\ (\text {S})$
EnMOGKSO vs. MOEA/D	$5.22 \times 10^{-5}\ (\text {S})$	$9.4 \times 10^{-6}\ (\text {S})$	$1.38 \times 10^{-5}\ (\text {S})$
EnMOGKSO vs. IMOMRFO	$1.01 \times 10^{-4}\ (\text {S})$	$1.13 \times 10^{-1}\ (\text {NS})$	$3.11 \times 10^{-4}\ (\text {S})$
EnMOGKSO vs. MOGTO	$2.38 \times 10^{-1}\ (\text {NS})$	$9.4 \times 10^{-6}\ (\text {S})$	$9.4 \times 10^{-6}\ (\text {S})$
EnMOGKSO vs. NSGA-II	$2.16 \times 10^{-1}\ (\text {NS})$	$1.52 \times 10^{-4}\ (\text {S})$	$3.39 \times 10^{-3}\ (\text {S})$
EnMOGKSO vs. MOGKSO	$9.39 \times 10^{-6}\ (\text {S})$	$9.4 \times 10^{-6}\ (\text {S})$	$6.38 \times 10^{-4}\ (\text {S})$

Table 10 shows that 27 of 33 pairwise comparisons are significant across HV, IGD, and PSP. The reported *p*-values and Friedman ranks jointly indicate both pairwise and overall differences across algorithms.

### Optimal WSN deployment results

#### Evaluation criteria

#### Spacing metric (SP)

SP measures how uniformly solutions are distributed on the Pareto front^67^; lower values indicate better diversity.

#### Mathematical definition

44 $$\begin{aligned} SP = \frac{1}{N - 1} \sqrt{\sum _{i=1}^{N-1} \left( d_i - \bar{d}\right) ^2} \end{aligned}$$

where $d_i$ is the distance between consecutive solutions, $\bar{d}$ is the mean distance, and *N* is the number of solutions on the front.

**Table 11:** SP evaluation (mean, best, worst, and SD) for different (*C*, *K*) settings.

C,k	Metric	EnMOGKSO	MOGKSO	MOGTO	MOPSO	MOSMA
C=1, k=1	Mean	3.89E+01	1.20E+02	6.27E+01	5.69E+01	7.68E+01
	Best	2.01E+01	1.02E+02	4.34E+01	3.99E+01	6.18E+01
	Worst	7.41E+01	1.56E+02	9.08E+01	7.04E+01	9.48E+01
	SD	1.84E+01	2.05E+01	1.75E+01	1.23E+01	1.42E+01
C=1, k=2	Mean	4.17E+01	1.22E+02	5.62E+01	4.29E+01	8.55E+01
	Best	1.70E+01	9.52E+01	2.72E+01	2.98E+01	7.17E+01
	Worst	9.89E+01	1.63E+02	9.40E+01	5.97E+01	1.00E+02
	SD	2.93E+01	2.32E+01	2.37E+01	1.32E+01	1.10E+01
C=1, k=3	Mean	4.44E+01	1.14E+02	5.94E+01	5.78E+01	9.13E+01
	Best	1.34E+01	8.67E+01	4.81E+01	4.01E+01	7.55E+01
	Worst	1.27E+02	1.45E+02	7.13E+01	7.97E+01	1.40E+02
	SD	4.45E+01	2.60E+01	9.75E+00	1.67E+01	2.47E+01
C=2, k=1	Mean	4.84E+01	1.06E+02	5.21E+01	4.70E+01	9.62E+01
	Best	2.84E+01	8.00E+01	4.23E+01	2.26E+01	6.25E+01
	Worst	9.75E+01	1.37E+02	6.13E+01	6.13E+01	1.42E+02
	SD	2.61E+01	2.06E+01	6.84E+00	1.36E+01	2.63E+01
C=2, k=2	Mean	2.83E+01	1.31E+02	6.29E+01	5.09E+01	8.24E+01
	Best	1.57E+01	1.01E+02	4.94E+01	3.41E+01	6.49E+01
	Worst	4.91E+01	1.76E+02	9.64E+01	9.01E+01	1.11E+02
	SD	1.30E+01	2.60E+01	1.88E+01	2.03E+01	1.79E+01
C=2, k=3	Mean	3.34E+01	1.11E+02	5.96E+01	5.00E+01	7.45E+01
	Best	1.31E+01	7.72E+01	3.40E+01	3.17E+01	6.35E+01
	Worst	4.44E+01	1.38E+02	9.87E+01	7.25E+01	9.21E+01
	SD	1.11E+01	2.39E+01	2.15E+01	1.91E+01	9.92E+00
C=3, k=1	Mean	4.08E+01	1.04E+02	5.07E+01	4.71E+01	8.19E+01
	Best	9.61E+00	8.21E+01	3.90E+01	2.85E+01	6.38E+01
	Worst	8.33E+01	1.31E+02	6.68E+01	6.42E+01	1.16E+02
	SD	2.71E+01	1.90E+01	1.05E+01	1.51E+01	1.99E+01
C=3, k=2	Mean	3.61E+01	1.15E+02	1.22E+02	4.60E+01	6.78E+01
	Best	1.50E+01	9.63E+01	6.13E+01	3.18E+01	5.23E+01
	Worst	6.56E+01	1.41E+02	2.12E+02	6.44E+01	8.70E+01
	SD	2.01E+01	1.74E+01	7.11E+01	1.36E+01	1.13E+01
C=3, k=3	Mean	4.86E+01	1.12E+02	5.55E+01	4.38E+01	8.48E+01
	Best	2.74E+01	7.62E+01	4.04E+01	2.90E+01	7.17E+01
	Worst	7.20E+01	1.53E+02	6.91E+01	5.48E+01	1.07E+02
	SD	1.64E+01	3.00E+01	1.11E+01	9.75E+00	1.32E+01

Table 11 shows the best SP behavior for EnMOGKSO across most (*c*, *k*) settings, with MOPSO usually second.

#### Performance evaluation of the EnMOGKSO-based deployment method

**Fig. 13:**
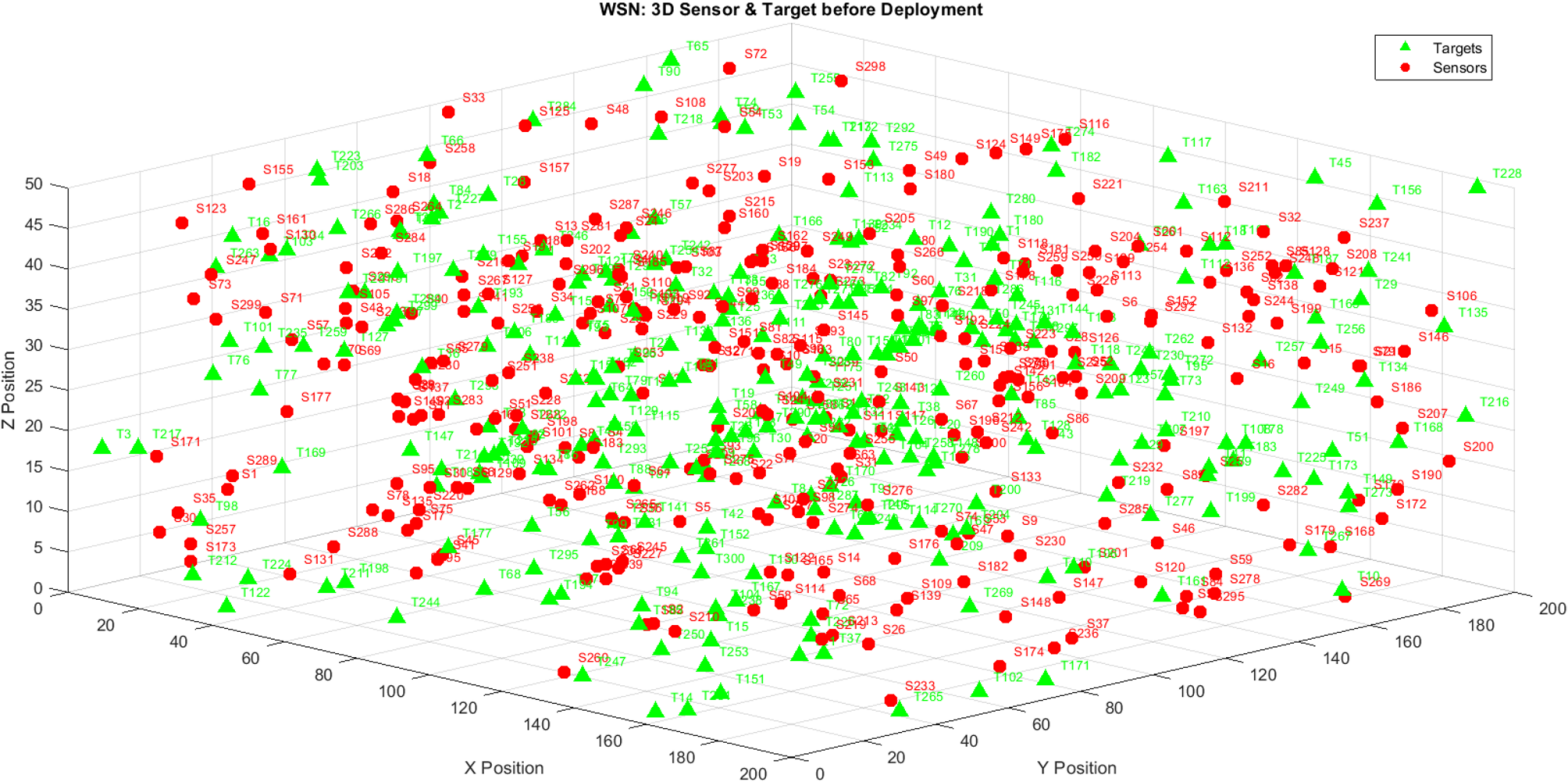
Initial 3D spatial distribution of sensors and targets in the WSN deployment space.

Figure 13 shows the initial 3D sensor-target layout.

The deployment study jointly optimizes *k*-coverage, *C*-connectivity, and cost in the same 3D environment, comparing MOGKSO, EnMOGKSO, MOPSO, MOGTO, and MOSMA.

**Table 12:** Coverage, connectivity, and cost metrics for all algorithms across all (*c*, *k*) settings.

C, K	Objectives	Metric	MOGKSO	EnMOGKSO	MOPSO	MOGTO	MOSMA
C=1, K=1	Coverage degree	Mean	6	11	11	7	5
		SD	4.8	1.56	1.63	4.21	4.24
	Connectivity degree	Mean	4	7	7	6	4
		SD	2.38	0.07	0.08	0.69	2.21
	Cost	Mean	10546	19396	19126	13422	10320
		SD	7898.21	1808.46	1721.22	5216.76	7474.44
C=1, K=2	Coverage degree	Mean	6	12	11	8	6
		SD	4.65	1.01	1.54	3.47	4.12
	Connectivity degree	Mean	5	7	7	7	4
		SD	2.31	0.02	0.1	0.15	1.96
	Cost	Mean	12111	19670	19501	15194	10522
		SD	7035.49	1305.72	1538.22	3864.03	7078.38
C=1, K=3	Coverage degree	Mean	6	11	11	8	6
		SD	4.7	1.46	1.87	3.46	3.83
	Connectivity degree	Mean	5	7	7	7	4
		SD	2.07	0.05	0.1	0.14	2.06
	Cost	Mean	10911	19391	19098	15148	11298
		SD	7220.41	1411.65	2018.79	4129.97	6656.06
C=2, K=1	Coverage degree	Mean	6	12	11	7	6
		SD	4.5	0.91	1.84	4.02	3.91
	Connectivity degree	Mean	5	7	7	7	4
		SD	2.16	0.13	0.1	0.16	1.95
	Cost	Mean	10956	19884	19452	14281	11298
		SD	7542.51	1146.12	1841.41	4863.33	6630.05
C=2, K=2	Coverage degree	Mean	6	11	12	8	6
		SD	4.78	0.87	1.6	4.04	4.21
	Connectivity degree	Mean	5	7	7	7	4
		SD	2.31	0.13	0.1	0.13	2.01
	Cost	Mean	11884	19395	19475	15919	11047
		SD	8113.22	1159.47	1974.11	4855.43	7183.61
C=2, K=3	Coverage degree	Mean	6	12	11	7	6
		SD	4.38	0.82	1.67	4	4.2
	Connectivity degree	Mean	5	7	7	6	4
		SD	2.02	0.02	0.07	0.26	2.06
	Cost	Mean	11925	19702	19744	14346	10511
		SD	6565.55	981.11	1790.66	4569.14	7222.49
C=3, K=1	Coverage degree	Mean	6	11	11	8	5
		SD	4.45	1.17	1.9	3.37	3.83
	Connectivity degree	Mean	5	7	7	7	4
		SD	2.2	0.05	0.11	0.15	1.93
	Cost	Mean	12047	19221	19459	15138	9506
		SD	7210.54	1380.37	1922.07	4086.29	7123.75
C=3, K=2	Coverage degree	Mean	7	12	11	7	6
		SD	4.34	1.01	1.54	3.85	3.812
	Connectivity degree	Mean	5	7	7	7	4
		SD	2.31	0.13	0.1	0.13	2.01
	Cost	Mean	12899	19304	18978	14251	10608
		SD	7234.85	832.81	1664.73	4436.48	6597.84
C=3, K=3	Coverage degree	Mean	6	12	11	7	5
		SD	4.74	1.22	1.6	3.46	4.08
	Connectivity degree	Mean	5	7	7	7	4
		SD	2.19	0.09	0.08	0.1	2.18
	Cost	Mean	11077	19319	19037	14879	10438
		SD	7401.22	1043.11	1822.56	4275.66	7076.94

Table 12 reports higher and more stable coverage and connectivity for EnMOGKSO (often with MOPSO), at higher cost (typically around 19k).

**Fig. 14:**
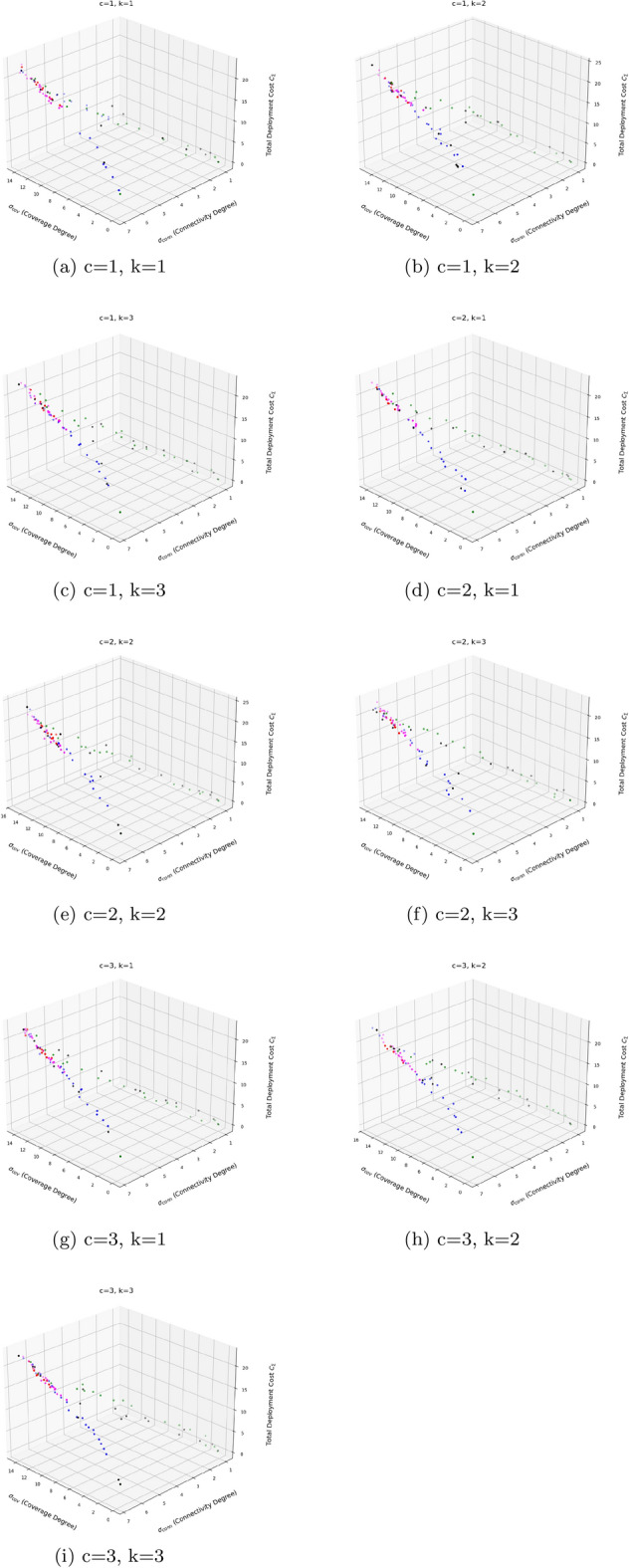
EnMOGKSO objectives for different values of *c* and *k*.

**Fig. 15:**
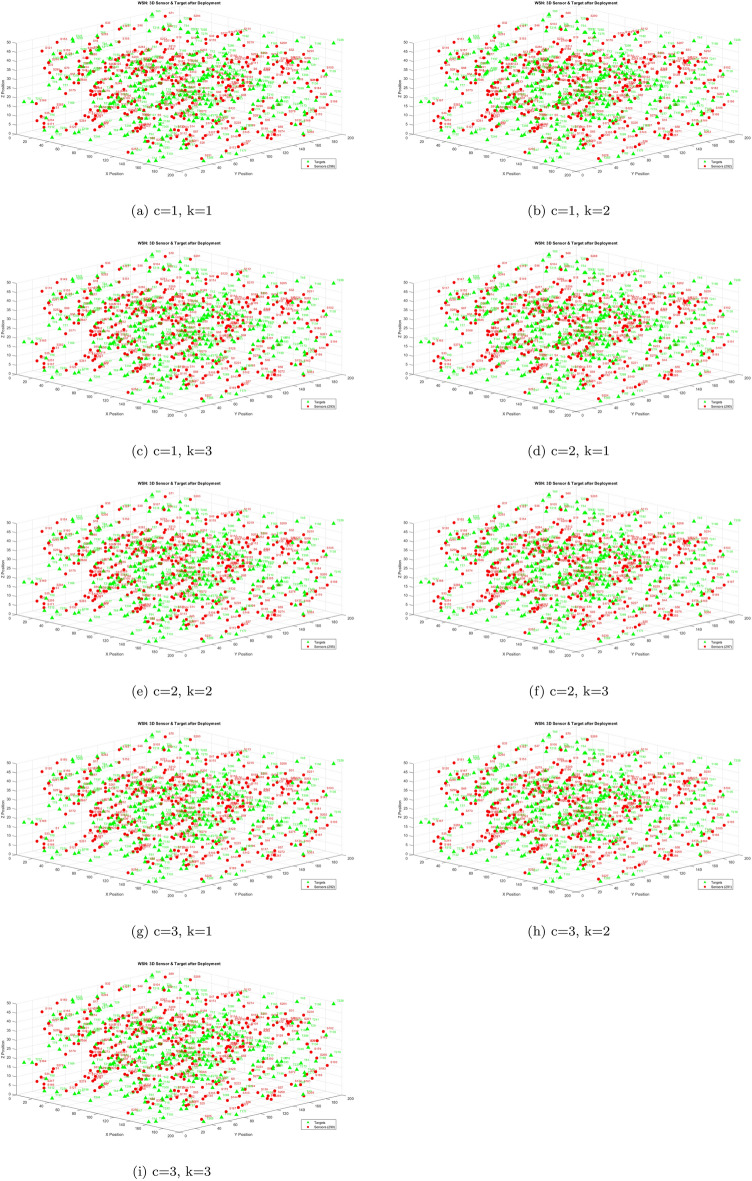
Deployment outcomes for different combinations of *c* and *k*.

Figures 14 and 15 show that increasing *c*/*k* tightens feasibility and shifts trade-offs toward higher cost.

**Fig. 16:**
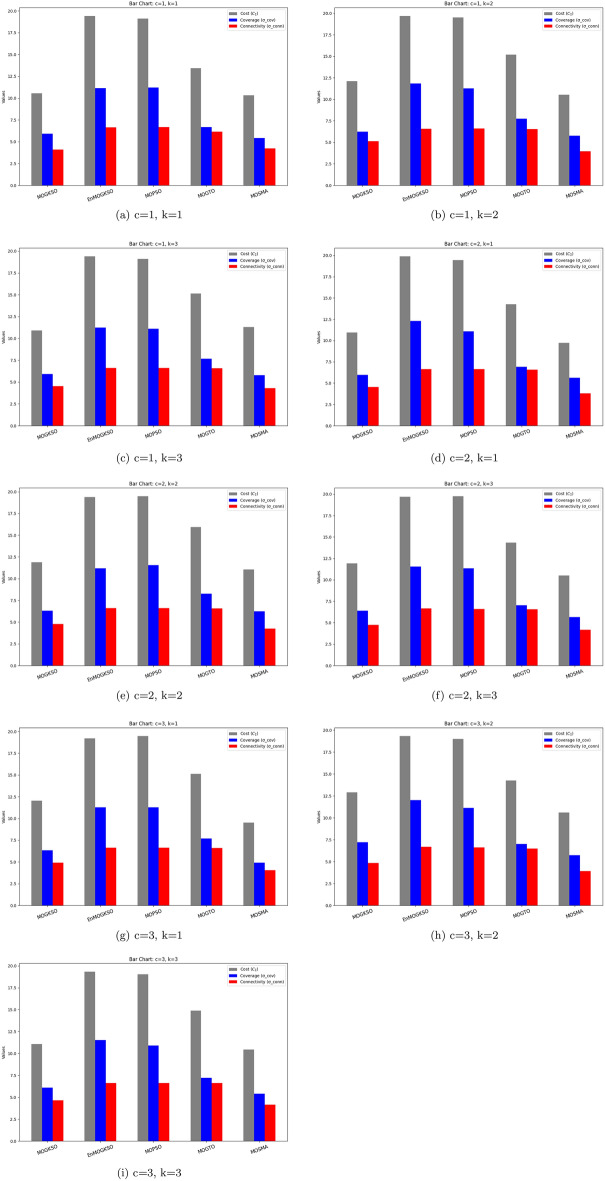
Bar-plot view of objective values for different combinations of *c* and *k*.

Figure 16 shows the same trade-off: EnMOGKSO and MOPSO provide higher quality at higher cost, while MOSMA has the lowest cost and the weakest quality.

**Fig. 17:**
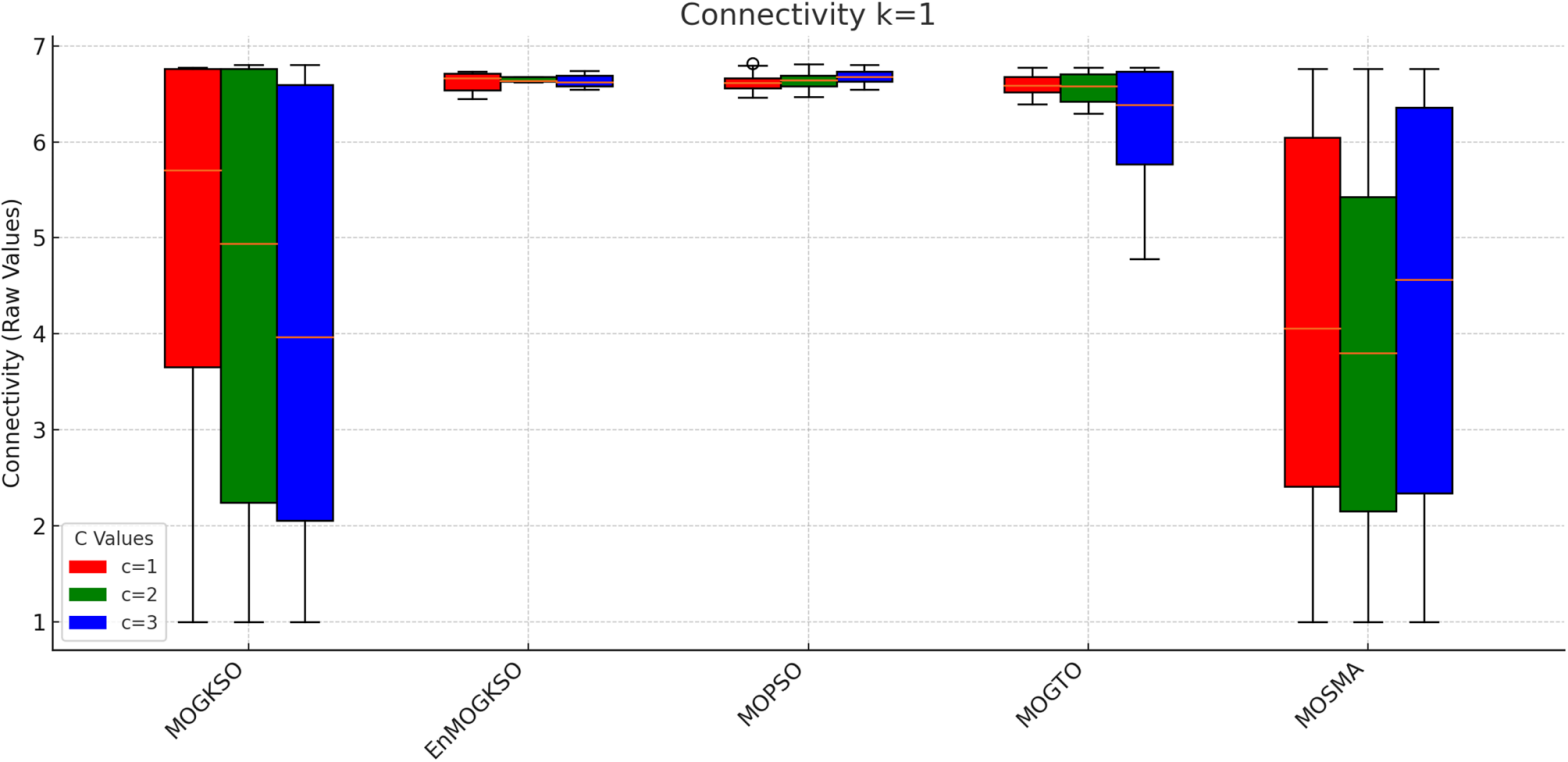
k=1.

**Fig. 18:**
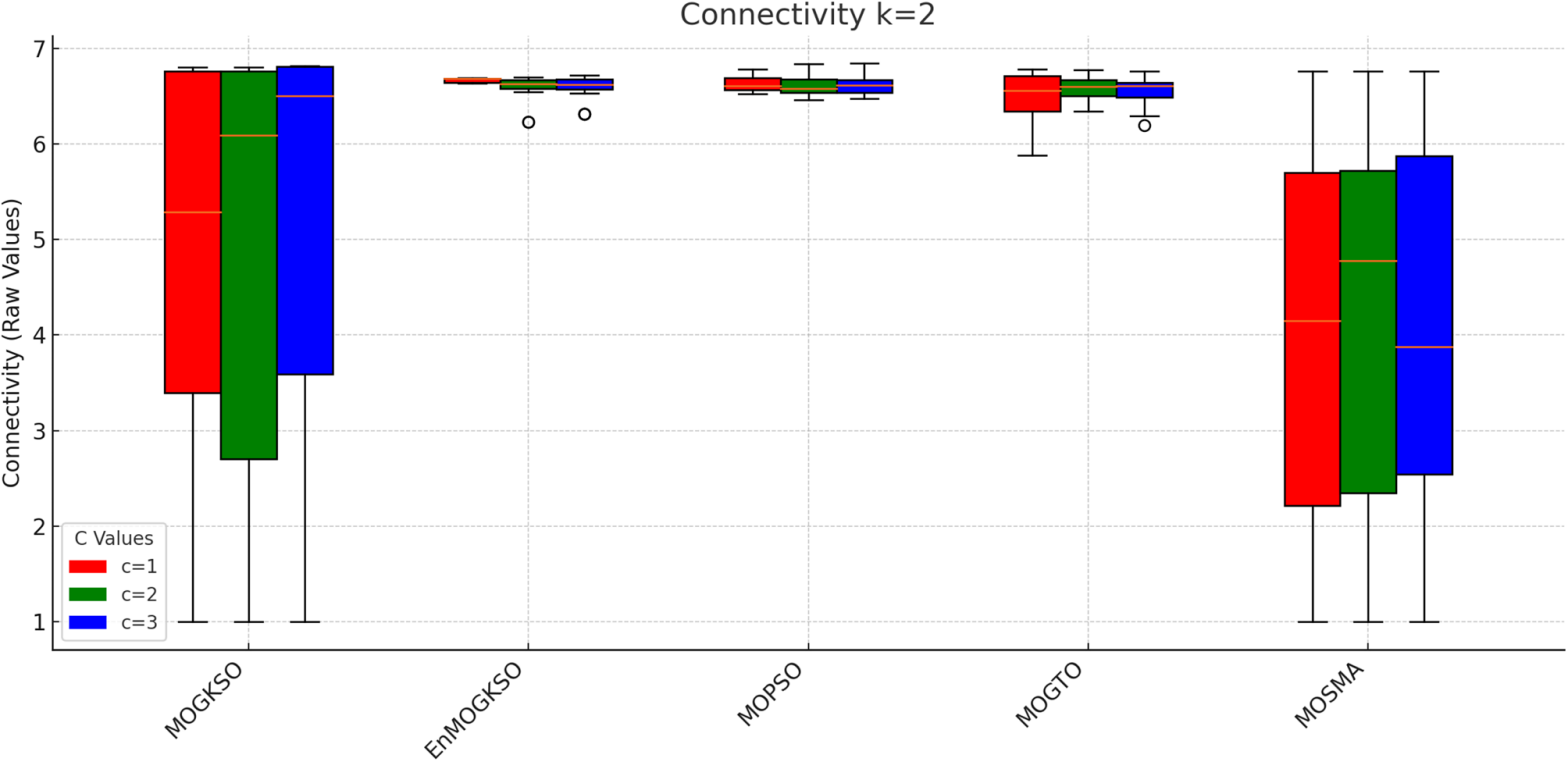
k=2.

**Fig. 19:**
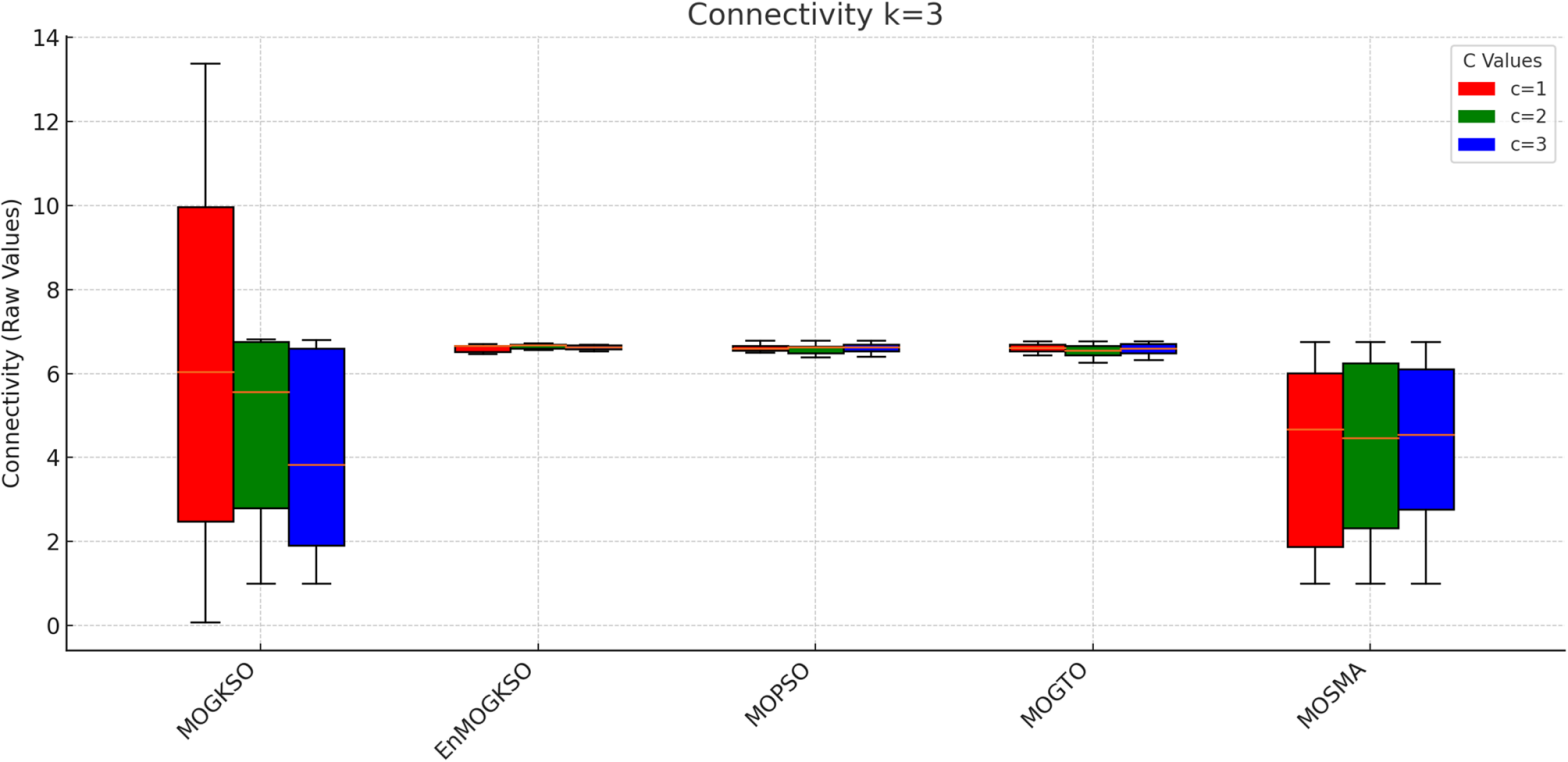
k=3.

**Fig. 20:**
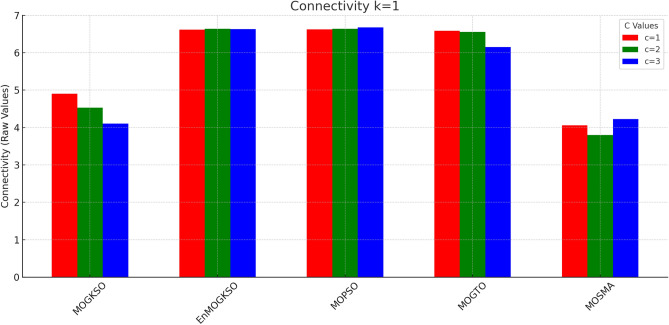
k=1.

**Fig. 21:**
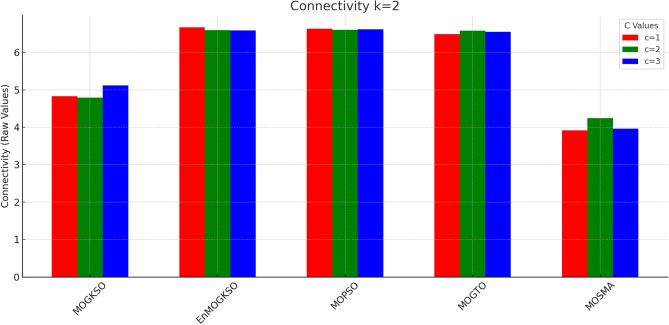
k=2.

**Fig. 22:**
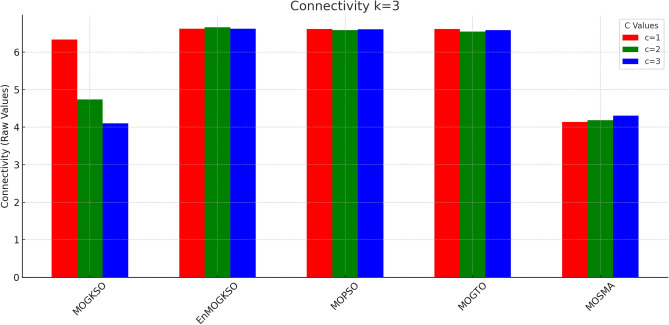
k=3.

Connectivity plots (Figs. 17, 18, 19, 20, 21, 22) place EnMOGKSO and MOPSO at the top, with a tighter spread for EnMOGKSO.

**Fig. 23:**
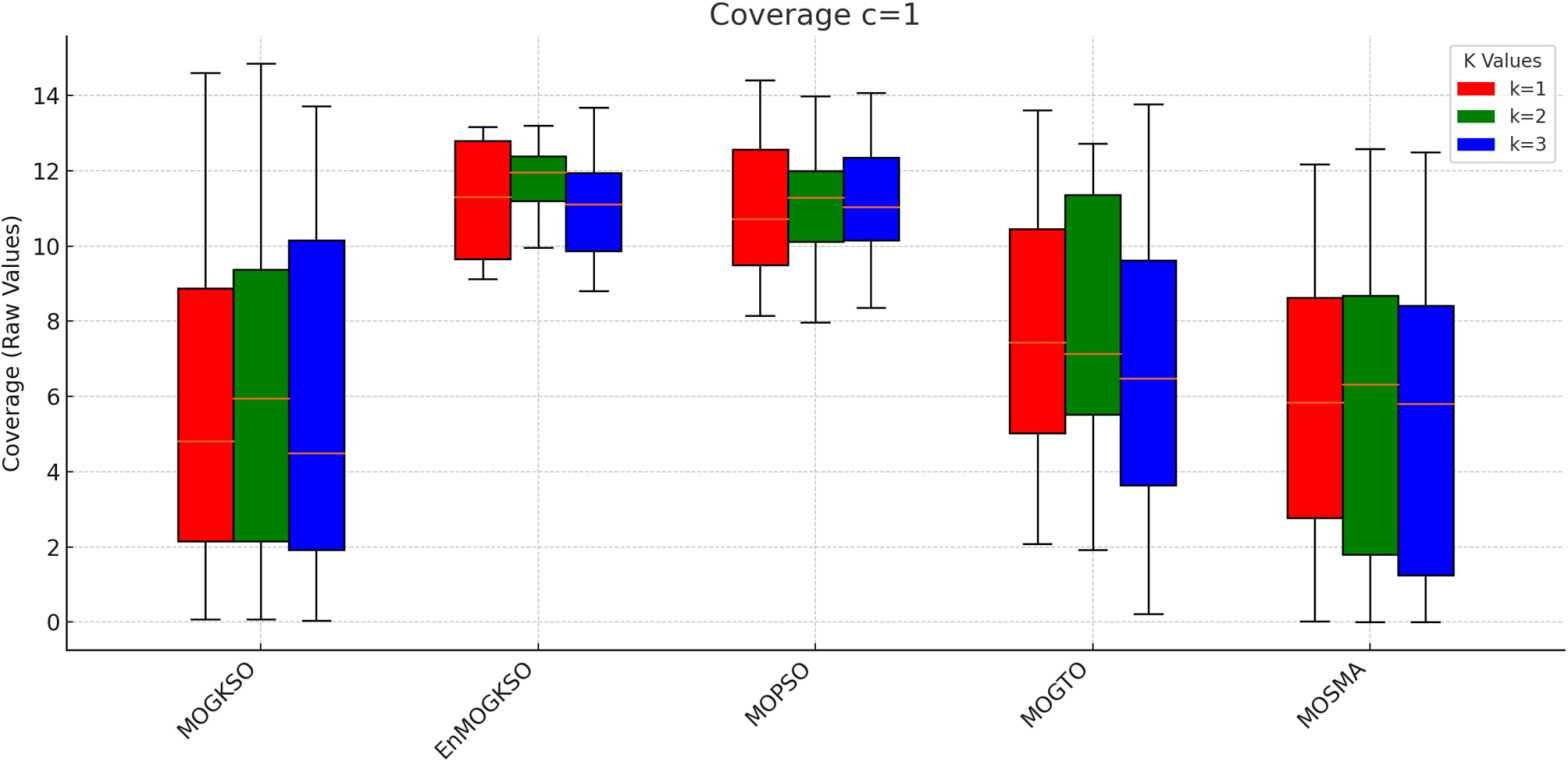
c=1.

**Fig. 24:**
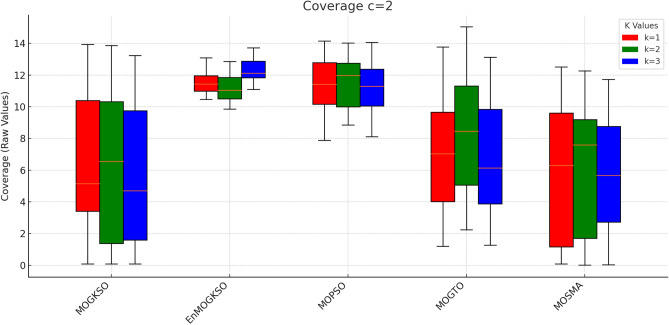
c=2.

**Fig. 25:**
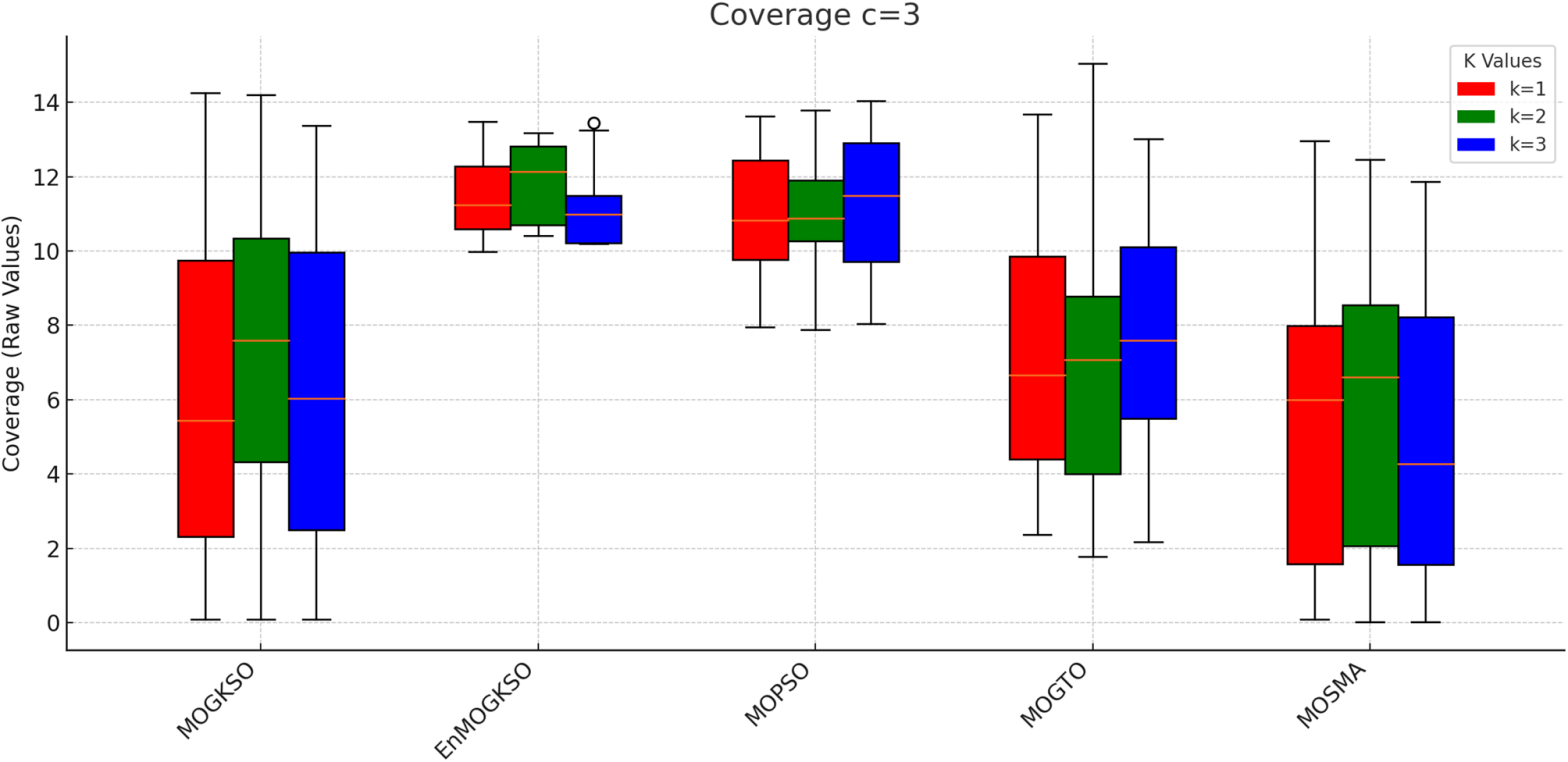
c=3.

**Fig. 26:**
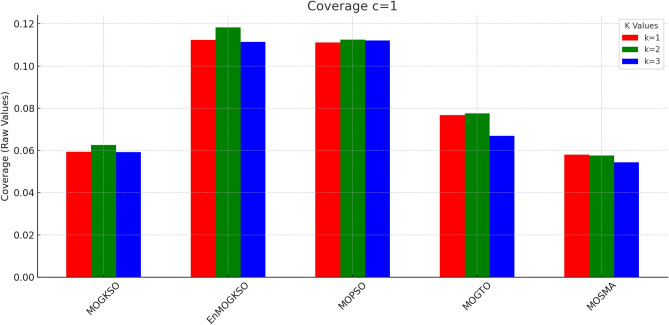
c=1.

**Fig. 27:**
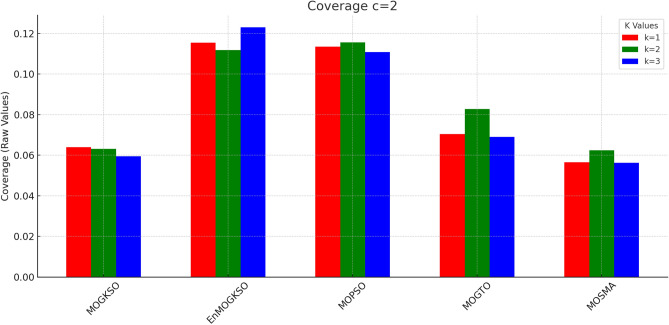
c=2.

**Fig. 28:**
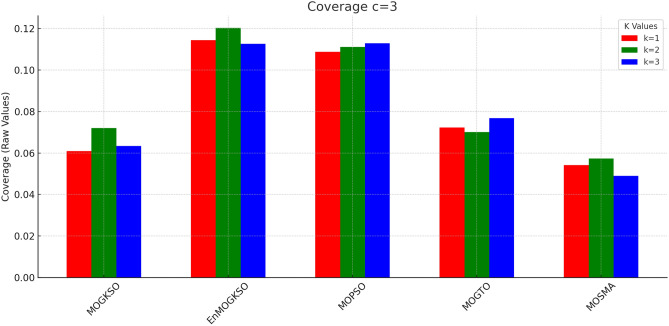
C=2.

Figures 23, 24, 25, 26, 27, 28 show dominant coverage for EnMOGKSO and MOPSO, again with tighter dispersion for EnMOGKSO.

**Fig. 29:**
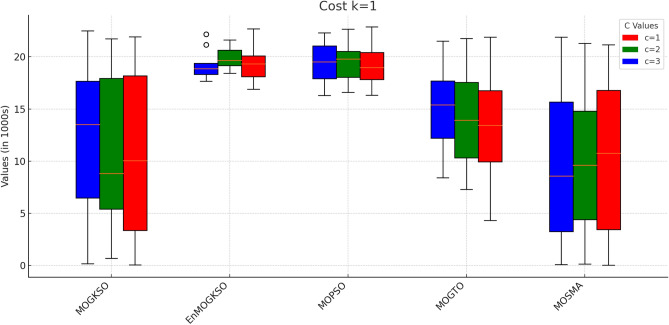
k=1.

**Fig. 30:**
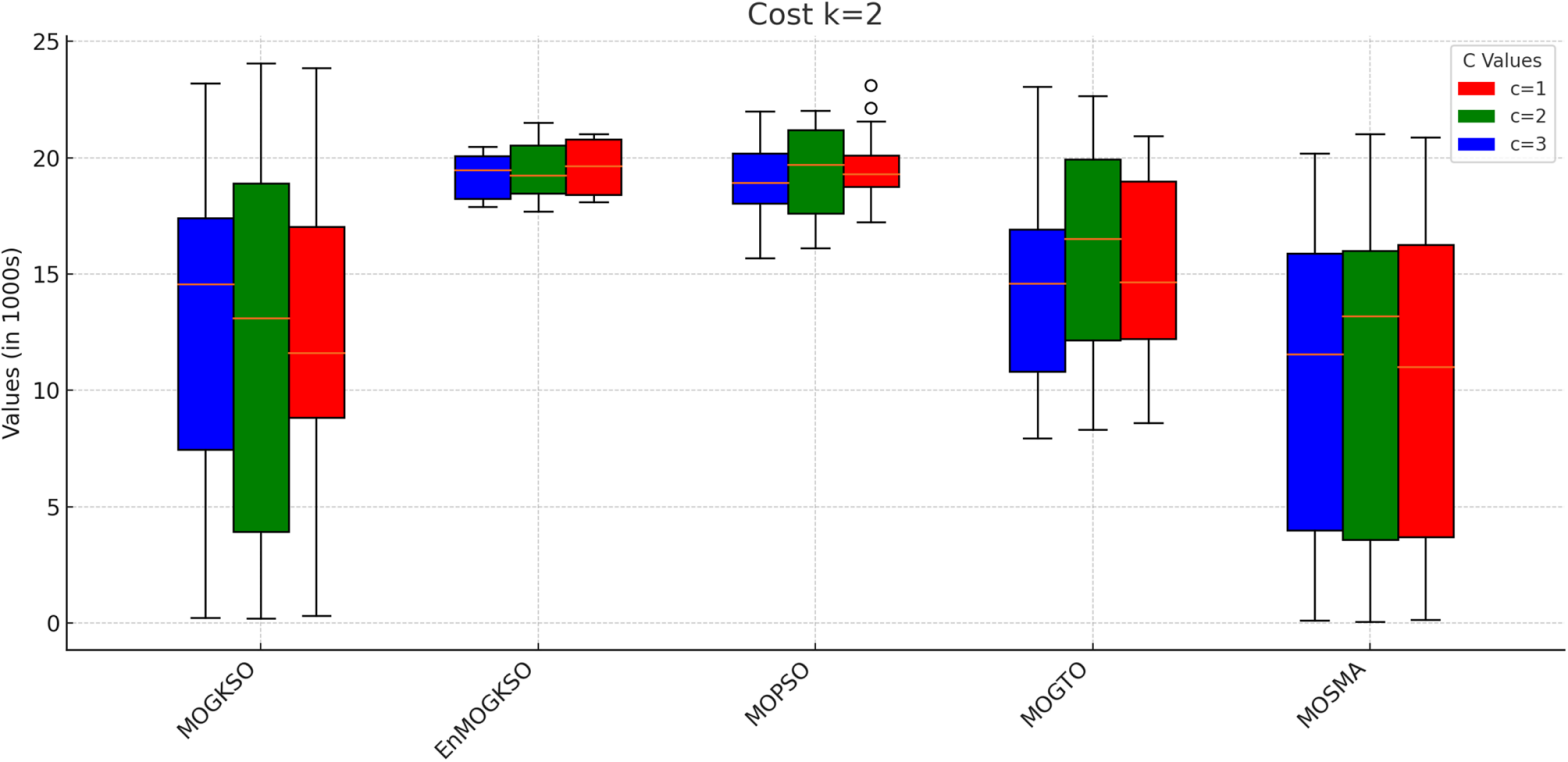
k=2.

**Fig. 31:**
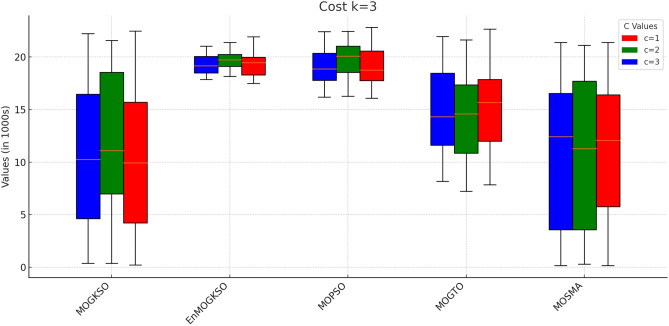
k=3.

**Fig. 32:**
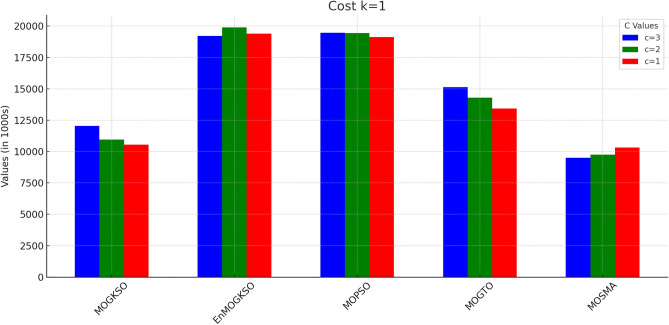
k=1.

**Fig. 33:**
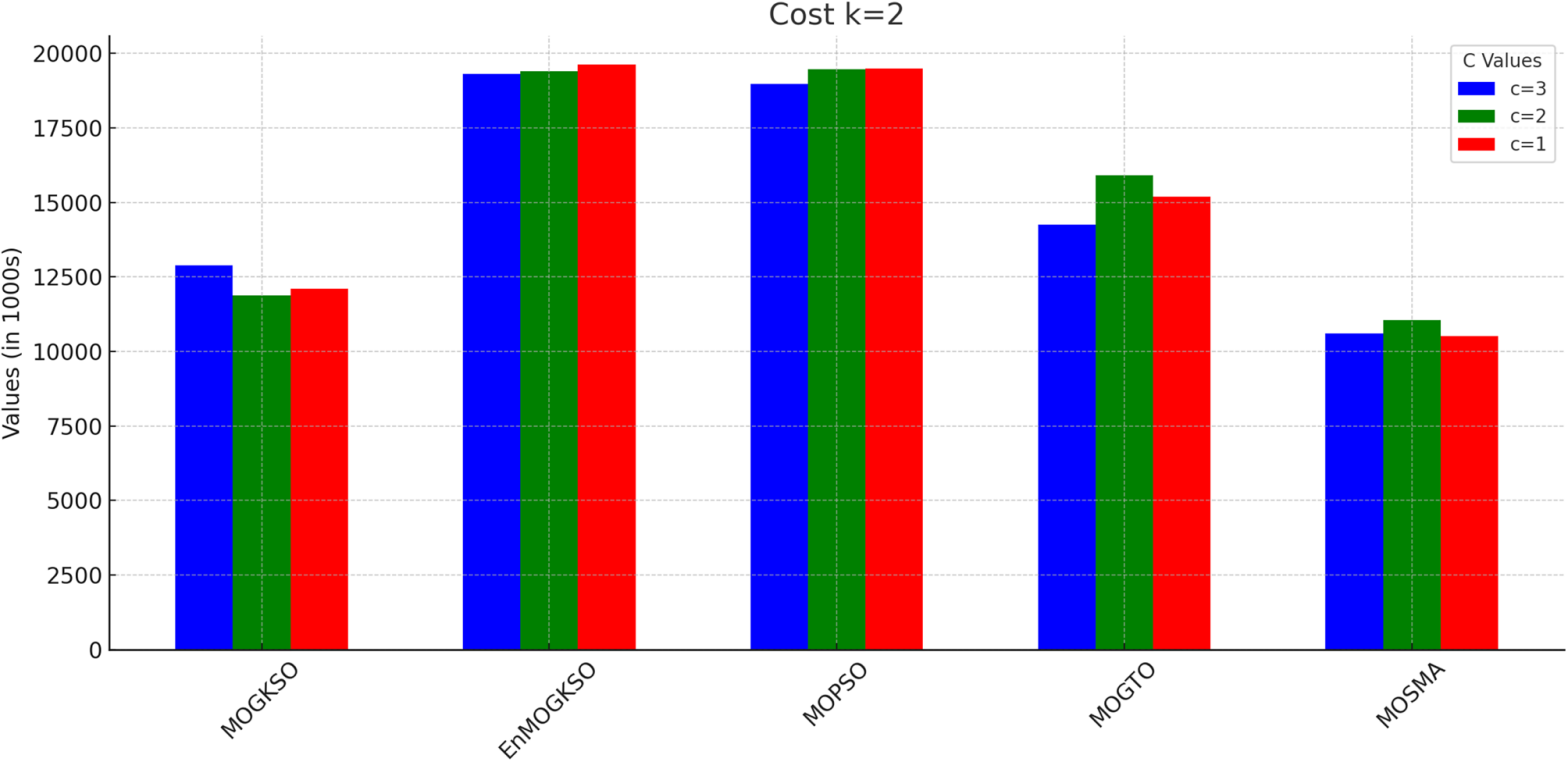
k=2.

**Fig. 34:**
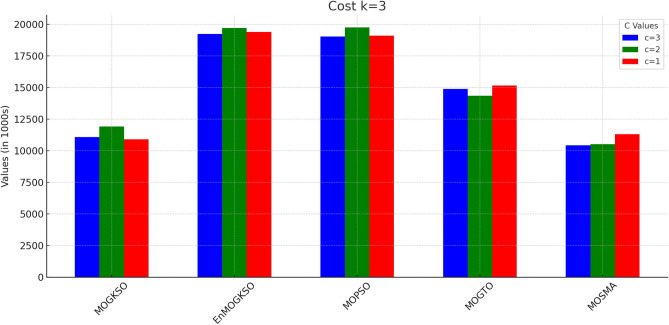
k=3.

Figures 29, 30, 31, 32, 33, 34 confirm the expected quality-cost trade-off.

**Table 13:** SP metrics for all algorithms across all (*c*, *k*) settings.

c, k	Metric	MOGKSO	EnMOGKSO	MOPSO	MOGTO	MOSMA
C=1, K=1	Mean	4.59E+02	0.00E+00	2.22E+02	4.01E+02	4.57E+02
	SD	1.08E+02	0.00E+00	1.17E+02	7.32E+01	1.34E+02
	Best	2.97E+02	0.00E+00	1.14E+02	2.93E+02	3.21E+02
	Worst	5.80E+02	0.00E+00	3.90E+02	5.15E+02	6.63E+02
C=2, K=1	Mean	5.97E+02	1.18E+01	1.57E+02	2.95E+02	4.94E+02
	SD	1.22E+02	2.88E+01	5.72E+01	5.62E+01	1.06E+02
	Best	4.24E+02	0.00E+00	7.46E+01	2.25E+02	3.33E+02
	Worst	7.30E+02	7.05E+01	2.41E+02	3.84E+02	6.59E+02
C=3, K=1	Mean	5.45E+02	1.93E+01	2.47E+02	3.56E+02	4.29E+02
	SD	1.37E+02	2.97E+01	1.37E+02	5.35E+01	1.46E+02
	Best	3.08E+02	0.00E+00	1.41E+02	2.95E+02	2.08E+02
	Worst	6.68E+02	7.57E+01	4.33E+02	4.46E+02	6.47E+02
C=1, K=2	Mean	6.38E+02	0.00E+00	1.75E+02	3.16E+02	5.59E+02
	SD	1.21E+02	0.00E+00	6.07E+01	6.24E+01	1.01E+02
	Best	5.00E+02	0.00E+00	1.01E+02	2.59E+02	4.92E+02
	Worst	8.54E+02	0.00E+00	2.74E+02	4.32E+02	7.53E+02
C=2, K=2	Mean	6.40E+02	4.02E+00	1.93E+02	3.57E+02	4.68E+02
	SD	1.66E+02	9.84E+00	5.51E+01	8.38E+01	8.79E+01
	Best	3.69E+02	0.00E+00	1.21E+02	2.50E+02	3.48E+02
	Worst	8.13E+02	2.41E+01	2.82E+02	4.71E+02	5.94E+02
C=3, K=2	Mean	5.53E+02	3.94E+00	1.97E+02	3.40E+02	5.36E+02
	SD	8.12E+01	9.65E+00	4.27E+01	9.72E+01	3.58E+02
	Best	4.43E+02	0.00E+00	1.58E+02	2.02E+02	6.70E+01
	Worst	6.71E+02	2.36E+01	2.74E+02	4.94E+02	4.78E+02
C=1, K=3	Mean	5.01E+02	5.82E+00	2.35E+02	3.69E+02	4.98E+02
	SD	7.50E+01	1.43E+01	5.60E+01	1.12E+02	6.12E+01
	Best	4.17E+02	0.00E+00	1.59E+02	2.29E+02	4.11E+02
	Worst	6.14E+02	3.49E+01	3.04E+02	4.89E+02	5.94E+02
C=2, K=3	Mean	6.54E+02	1.74E+01	1.96E+02	3.39E+02	3.87E+02
	SD	1.04E+02	2.98E+01	4.90E+01	7.29E+01	9.29E+01
	Best	5.02E+02	0.00E+00	1.04E+02	2.19E+02	2.20E+02
	Worst	7.47E+02	7.25E+01	2.35E+02	4.01E+02	4.80E+02
C=3, K=3	Mean	6.48E+02	1.37E+01	1.81E+02	3.67E+02	4.82E+02
	SD	1.83E+02	3.36E+01	4.47E+01	1.25E+02	8.72E+01
	Best	3.70E+02	0.00E+00	1.40E+02	1.92E+02	4.16E+02
	Worst	9.02E+02	8.22E+01	2.66E+02	5.76E+02	6.42E+02

Table 13 corroborates Table 11: EnMOGKSO yields the smallest SP values in most (*c*, *k*) settings.

**Table 14:** Algorithm runtimes for different (*k*, *c*) settings (in $10^3$ seconds).

K	C	MOGKSO	EnMOGKSO	MOPSO	MOGTO	MOSMA
1	1	10.56	11.89	109.8	9.43	9.44
1	2	10.30	11.04	116.1	9.31	9.37
1	3	10.47	11.44	113.9	9.54	9.36
2	1	10.15	11.48	125.6	9.73	9.52
2	2	10.99	11.12	113.0	9.75	9.42
2	3	10.72	11.04	115.0	9.53	9.26
3	1	10.42	11.08	116.5	9.65	9.47
3	2	10.76	11.08	114.8	9.39	9.28
3	3	10.60	11.26	108.8	9.28	9.26

Table 14 shows moderate runtime overhead for EnMOGKSO relative to lower-cost baselines.

## Conclusion and future work

EnMOGKSO shows competitive and stable performance on CEC 2020 and the heterogeneous 3D WSN deployment task, with improved convergence-diversity balance under constrained settings.

Despite the improvements introduced in EnMOGKSO, several limitations remain that provide directions for future research. First, although mechanism-level analyses and partial operator studies are presented, a comprehensive component-wise ablation of all enhancement mechanisms under strictly controlled computational budgets remains limited and should be further investigated. Second, the current study focuses on a three-objective deployment formulation; extending the framework to many-objective optimization settings may require additional diversity preservation strategies and archive management mechanisms. Third, the experiments are conducted under static deployment assumptions. In practical Internet-of-Things (IoT) and WSN environments, dynamic conditions such as node failures, environmental changes, or time-varying coverage demands may require adaptive or online optimization strategies.

Future work will therefore focus on developing stronger adaptive parameter control mechanisms, extending the algorithm to many-objective and dynamic deployment scenarios, and conducting more extensive ablation analyses under matched computational budgets. Additionally, large-scale real-world deployment studies will be explored to evaluate the robustness, feasibility, and scalability of the proposed framework when applied to practical sensing infrastructures where coverage reliability, communication stability, and cost efficiency must be maintained simultaneously.

## Data Availability

The datasets used and/or analysed during the current study available from the corresponding author on reasonable request.
